# Asymmetry in reproduction strategies drives evolution of resistance in biological control systems

**DOI:** 10.1371/journal.pone.0207610

**Published:** 2018-12-19

**Authors:** Paula Casanovas, Stephen L. Goldson, Jason M. Tylianakis

**Affiliations:** 1 Bio-Protection Research Centre, School of Biological Sciences, University of Canterbury, Christchurch, New Zealand; 2 Bio-Protection Research Centre, Lincoln University, Lincoln, New Zealand; 3 AgResearch Ltd., Christchurch, New Zealand; 4 Department of Life Sciences, Imperial College London, Silwood Park Campus, Ascot, Berkshire, United Kingdom; University of Minnesota, UNITED STATES

## Abstract

The success of biological control may depend on the control agent co-evolving with its target pest species, precluding the emergence of resistance that often undermines chemical control. However, recent evidence of a decline in attack rates of a sexual pest weevil by its asexual parasitoid suggests that evolutionary arms races may not prevent the emergence of resistance if the host and parasitoid do not have reproductive strategies that generate equal amounts of genetic variation. To understand how these asymmetries in reproductive strategies may drive the emergence of resistance, we combined life history data from two pest weevils and their parasitoids (one sexual and one asexual) in the New Zealand pastoral ecosystem, with a population dynamic model that allows the coevolution of hosts and parasitoids. We found that the ratio of the genetic variance of hosts to parasitoids was a key determinant of the emergence of resistance. Host resistance eventually occurred unless the parasitoids had considerably greater additive genetic variance than their host. The higher reproductive rate of asexual parasitoids did little to offset the cost of reduced additive genetic variance. The model predictions were congruent with long-term parasitism rates observed in the field for both of the pests considered (one with a sexual and one with an asexual parasitoid). We then explored the consequences of introducing two parasitoids with different reproductive strategies that attack the same sexual host. The model showed that the sexually reproducing parasitoid always out-competed the asexually reproducing one. Our study shows that any asymmetry in reproductive strategies is extremely important for predicting the long-term success of biological control agents. Fortunately, introduction of sexually reproducing individuals after an initial introduction of asexual strains may overcome the problems of host resistance. We conclude that evolution must be considered when evaluating the long-term outcomes of importation biological control.

## Introduction

Regulation of pesticides is increasing internationally, fuelled by concerns about impacts on human health and the environment [1]. Combined with the cost of rapid evolution of resistance to chemical insecticides [2], demand is growing for sustainable pest control strategies [3], including integrated pest management [4]. Biological control will likely form a component of such strategies, particularly if reduced pesticide use improves the survival of natural enemies. A key advantage of importation biological control (as described in [5]) is its long-term sustainability if initially successful [6]. Moreover, the evolution of pest resistance to natural enemies such as parasitoids is considered unlikely because an evolutionary arms race allows the enemy to evolve counter-adaptations [7–9]. In this sense, the success of a biological-control agent may depend on it co-evolving with the pest, rather than it being unresponsive to host adaptation [10]. In fact, a lack of sufficient co-evolutionary dynamics has been highlighted as one of the possible mechanisms by which successful biological control can break down [11]. A recent example of rapid decline in parasitism rates in a host-parasitoid system, has been shown in the dynamic between the Argentine stem weevil, *Listronotus bonariensis* (Kuschel) (Coleoptera: Curculionidae) and its asexual parasitoid *Microctonus hyperodae* (Loan) (Hymenoptera: Braconidae). This work points to the suggestion that arms races *per se* may not always prevent the emergence of resistance [12]. Specifically, it has been suggested that differences in additive genetic variance of the sexual weevil population versus its asexual parasitoid may have caused the emergence of apparent resistance [13]. Additive genetic variance is known to relate to the ability of a population to respond to selective pressures [14], and thus should be important in coevolution. The asymmetry in reproductive strategies between the host and parasitoid would be expected to generate differences in additive genetic variance, as populations of sexually reproducing organisms have greater additive genetic variance than those of asexual organisms [15]. Although asymmetries in additive genetic variance are well known in the case of rapidly-evolving pathogens and slowly reproducing hosts (Fenner 1983), to our knowledge they have not been considered in the context of biological control when pathogens are not the control agent [16].

In host-parasitoid biological control systems, there are many advantages associated with introducing an asexual parasitoid to control a given pest. In general, they have higher rates of population increase than sexual reproducers, during mass rearing there is no “waste” of hosts in the production of males [17], and they avoid Allee effects that can occur due to declining mate encounter rates [18]. Overall, asexual parasitoids are better colonizers than their sexual counterparts and, indeed, in some circumstances they are able to suppress a host population to a level lower than a comparable sexual form [18]. However, when hosts reproduce sexually and the control agent asexually, then the asymmetry in additive genetic variance could be detrimental for the biological control system in the longer term. Such asymmetry may stop the arms race that prevents the emergence of host resistance. In short, sexual forms of a parasitoid will, through genetic recombination, adapt at a faster rate to changed circumstances [19] than the asexual form; this includes the evolution of resistance by the host. In contrast, clonal lines of asexual parasitoids depend on mutations as the only source of genetic variability, and it is not clear whether any asexual advantages gained from initial greater host suppression are sufficient to offset this evolutionary disadvantage. For these reasons, it has even been suggested that asexual strains could be introduced first for rapid colonisation, followed by sexual strains to provide longer-term adaptive potential [18]. Although this proposition is intuitively logical, it remains unclear whether the benefits of this added adaptive potential would allow sexual strains to out-compete asexual lines that have a higher intrinsic population growth rate. More generally, it is unclear whether a greater number of parasitoid species or lines (asexual or sexual) inhibits the evolution of resistance by their hosts. Although biological control is essentially applied population biology, there are very few cases where population dynamic models have been used to provide guidance on the implementation of this practice [20], and to our knowledge there is no incorporation of evolutionary processes into these assessments.

Thus, this contribution seeks to understand how asymmetry in reproductive strategies of hosts and parasitoids relate to the emergence of resistance and the continuing success of biological control agents, and whether this asymmetry can be offset by the higher attack rates of asexual parasitoids. Although we were initially motivated by the asymmetric reproductive strategies (sexual versus asexual) generating differences in additive genetic variance, differences in additive genetic variance can also arise through other mechanisms (such as very small population bottlenecks and/or inbreeding). In order to explore these aspects, we used a coupled population dynamic model that allows the co-evolution of host resistance to parasitism and parasitoid “virulence” (Hochberg and Holt, 1995 [21]). As an illustrative case study, we used two examples of introduced pasture pest weevils in New Zealand that were, at least initially, controlled by introduced parasitoids. In these examples both hosts reproduce sexually, whereas in one case the parasitoid is asexual and in the other the parasitoid reproduces sexually. This aimed at shedding further light on the significance of reproductive asymmetry, and allowed us to ground-truth predictions of our model. We expected that sexually reproducing parasitoids would maintain higher parasitism rates than asexuality reproducing ones, providing more effective pest control. We used published empirical data on the species’ biologies to parameterize our models, as well as published long-term data on field parasitism rates to compare with model predictions.

We also extended Hochberg and Holt’s (1995) model to test for the effect of the inclusion of an additional parasitoid species into an already established host-parasitoid system. In particular, we asked if it was possible to rescue, enhance or disrupt an established biological control system, depending on the additive genetic variance of the initial host/parasitoid populations and that of a parasitoid introduced subsequently to the first system being established. Here, we expected that sexually reproducing parasitoids would be able to rescue unsuccessful biological control by asexually reproducing parasitoids. This, in turn, provided the opportunity to explore the role of parasitoid diversity in the emergence of host resistance.

## Materials and methods

### Model framework (“two-species model”)

We used a model originally developed by Hochberg and Holt (1995 [21]) to study the evolution of refuges (host unavailability for parasitism or resistance) alongside the population dynamics of coupled host-parasitoid associations for our two study systems. Throughout this work we call this first model a “two-species model”. It is important to note that the genetics of the host and parasitoid are not explicitly addressed in this model, and there is not tracking of gene frequencies or assumptions of dominance or recessiveness. Rather, the model considers co-evolutionary changes in quantitative characters that influence the proportion of hosts that is resistant to the parasitoid. The discrete, non-overlapping generation model is as follows:

$$N_{t+1}=\lambda \;N_{t}g\{N_{t}\}\;\left(\alpha +\left(1‑\alpha \right)f\{N_{t},P_{t}\}\right)$$ (1)

$$P_{t+1}=cN_{t}g\left\{N_{t}\right\}\;\left(1‑\alpha \right)\left(1‑f\{N_{t},P_{t}\}\right)$$ (2)

where N_t_ is the density of host (weevils) at the beginning of generation t, and P_t_ is the density of female parasitoids during generation t. The parameter λ represents the intrinsic growth rate of the weevil population; *c* is the mean number of parasitoid females surviving per parasitized host; and α is the proportion of weevils that is resistant to parasitism. We assumed that the weevil population is self-regulated when there are no parasitoids in the system, and we used the same density-dependent growth function *g*{N_t_} chosen by Hochberg and Holt (1995) to describe this:

$$g\left\{N_{t}\right\}={\left[1+N_{t}\;\left(\lambda -1\right)/K\right]}^{‑1}$$ (3)

where K is the ecological carrying capacity of the weevil population, and we assigned λ > 1 and K > 0 to ensure weevil population persistence in the absence of the parasitoid.

The proportion of susceptible weevils that escape parasitism is given by the function *f*{N_t_, P_t_}:

$$f\left\{N_{t},P_{t}\right\}=[1+(aP_{t}/k{\left(1+aN_{t}\;g\left\{N_{t}\right\}\left(1-\alpha \right)/\eta \right]}^{‑k}$$ (4)

where *a* is the parasitoid searching efficiency, *k* is the spatial heterogeneity in parasitism and η is the maximum number of weevils that each female parasitoid is able to parasitise (intrinsic attack rate).

The co-evolutionary integration of the model builds upon the above ecological model, and has three parts: the “resistance” functions, the “character” functions, and the “cost” functions. The model assumes that resistance and “virulence” depend on a single character in each species, designated respectively as n for the weevil and p for the parasitoid. The proportion of weevils resistant to parasitism is defined as a function of the average character value of both species. Therefore, the “resistance” function that determines the proportion of weevils resistant to parasitism is as follows:

$$\alpha_{t}\left\{\bar{n},\bar{p}\right\}=1‑exp\left\{‑{\left({\bar{n}}_{t}‑{\bar{p}}_{t}\right)}^{2}\right\}$$ (5A)

The “character” functions, which are the connection between the ecological model and its evolutionary component are as follows:

$${\bar{n}}_{t+1}={\bar{n}}_{t}+\Gamma_{n}\left[\partial ln\;W_{n}/\partial \bar{n}\right]$$ (6A)

$${\bar{p}}_{t+1}={\bar{p}}_{t}+\Gamma_{p}\left[\partial ln\;W_{p}/\partial \bar{p}\right]$$ (6B)

In these equations, Γ_n_ and Γ_p_ are the values for the additive genetic variance (a proxy for adaptive potential) of the weevil and the parasitoid respectively. Here it is important to note that we used these values to capture a key feature of the reproductive strategy of the parasitoids: if the parasitoid reproduces asexually with no possibility of adapting to its host (i.e. the value of the character will not change over time) and with that the additive genetic variance is equal to 0. This is of course the extreme case where we assumed that the rate of beneficial mutations in the parasitoid is negligible relative to the new phenotypes produced by recombination of available genetic variance in the weevils. In this study we also explored a range of cases for different ratios of additive genetic variance between the host and the parasitoid; here if the additive genetic variance is greater than 0, then the parasitoid reproduces sexually or accrues mutations and therefore has adaptive potential. As in Hochberg and Holt (1995) we assumed that selection pressure is weak so the additive genetic variance in the population is constant over time. We did not make any assumptions about the magnitude of the additive genetic variance, and also explored a wide range of possibilities in the model, as we do not have any data about the genetic variance in the modelled systems. W_n_ = N_t+1_/N_t_ and W_p_ = P_t+1_/P_t_ are the fitnesses of the weevils and the parasitoids, respectively.

Finally, the “cost” functions describe the cost of character evolution. An increase in the mean value of the character in the weevils incurs a direct cost ($C_{n}\bar{n}$) imposed on the otherwise cost-free intrinsic growth rate of the population λ*:

$$\lambda_{t}\left\{{\bar{n}}_{t}\right\}=\lambda *‑C_{n}{\bar{n}}_{t}$$ (7)

For the parasitoid, an increase in the character value incurs a direct cost ($C_{n}\bar{p}$) imposed on the otherwise cost-free intrinsic attack rate, η*:

$$\eta_{t}\left\{{\bar{p}}_{t}\right\}=\eta *‑C_{n}\;{\bar{p}}_{t}$$ (8)

Such costs of evolving resistance or virulence have been discussed widely in the literature (see [22,23] and references therein), and would occur if, for example, the host uses behavioural responses to avoid parasitism, which interfere with its ability to feed but also slow the parasitoid’s attack rate. Even though we could express the cost of character change using other parameters such as the searching efficiency (a) or the within-host survival rate (c), here we use η following the original model of Hochberg and Holt (1995) because this parameter can be interpreted in the same unit as λ (offspring produced). Complete information on the development of the two-species model and exploration of parameter space can be found in the detailed work of Hochberg and Holt (1995).

### Model parameterization and study cases

There were no data available to parameterize the evolutionary component of the models, so we used the parameter values suggested by Hochberg and Holt (1995) in their original model. We parameterized the ecological part of the model using data from the literature (Table 1) for two study systems in New Zealand’s simplified grassland ecosystems.

**Table 1:** Parameter values for the different biological control systems in this study and the range of values for each parameter explored during simulations.

Parameter	Definition	*L*. *bonariensis* - *M*. *hyperodae*	*S*. *discoideus -* *M*. *aethiopoides* (Moroccan strain)	Range of values used for analysis
λ	Intrinsic growth rate of the host	1–18	6–91	1–100
K	Host carrying capacity	720	773	50–800
η	Intrinsic rate of attack of the parasitoid	42±22	33	20–64
c	Survival of parasitoid larvae	0.92	?	0.5–1
κ	Spatial heterogeneity in parasitism	0.23–0.87	0.11	0.1–0.9
a	Searching efficiency	4.14	?	1–8
г_n_	Additive genetic variance in host	0.01	0.01	0–0.1
г_p_	Additive genetic variance in parasitoid	0	0.01	0–0.1
C_n_	Cost to host character	1	1	0–2
C_p_	Cost to parasitoid character	-	1	0–2
N_0_	Initial host density	K	K	50–800
P_0_	Initial parasitoid density	10	10	1–100
n_0_	Initial host character	1	1	0.5–1.5
p_0_	Initial parasitoid character	0.9	0.9	0.5–1.5

See S1 File for references to support these values and their units.

The first system involved the Argentine stem weevil, *Listronotus bonariensis* (Kuschel), which was first recorded in New Zealand in 1927. It is generally accepted that this species was probably accidentally introduced around the turn of the twentieth century [24]. The weevil can cause severe damage to the very important ryegrass component of New Zealand’s extensive areas of improved pasture. In 1991 the thelykotous (i.e. females produce clonal offspring via unfertilised eggs) braconid endoparasitoid *Microctonus hyperodae* (Loan) was introduced with the aim of suppressing this species [25]. This biological control agent was successful for the first seven years, however, its effectiveness began to decline thereafter [12]. The heritability of these reduced parasitism rates in the laboratory [26] suggests that they may have arisen through the evolution of resistance by the Argentine stem weevil population, with no, or a slower, co-evolutionary response from the asexual wasp.

The second system, the lucerne weevil, *Sitona discoideus* Gyllenhal (Coleoptera: Curculionidae), was discovered in New Zealand in 1974. It was probably introduced from Australia, but it originates from the Mediterranean area [27]. The damage resulting from larval feeding on rhizobial root nodules led to yield losses of up to 43% in young lucerne stands [28]. In 1982 a sexually reproducing Moroccan strain of the parasitoid *Microctonus aethiopoides* Loan (Hymenoptera: Braconidae) was introduced as a control agent [29]. After the introduction of this parasitoid, the observed levels of parasitism were subsequently found to be sufficient to offset economic impacts [30] such that insecticidal control was usually found to be unnecessary. These parasitism levels have been maintained over time.

**Population persistence**, **parasitism rates** and the **proportion of resistant hosts** were model outputs and we did not use data derived from the outputs for the parameterization of the model. Information supporting the values shown in Table 1 is found in S1 File.

### Sensitivity analysis

To investigate the evolution of resistance as a function of evolutionary asymmetries in the two-species model, we began by performing a sensitivity analysis to study the influence of each parameter in terms of the model output (i.e. the proportion of resistant hosts (α_t_) at t = 300 generations). Thus we calculated partial rank correlation coefficients to establish how strong the linear associations are between the result (proportion of resistant hosts) and each input parameter, after removing the linear effect of the other parameters [31]. We used the Latin-Hypercube sampling technique [31] to obtain values of the parameters from a uniform distribution with minimum and maximum values. These are described in Table 1 under the column headed “range of values used for analysis”. Note that, wherever possible, we used a range of parameter values that were obtained from the literature (Table 1). We generated 6000 parameter combinations (and samples of the model results), and calculated the partial rank correlation coefficients from these samples (any sampling size above 2000 gave qualitatively similar partial rank correlations, see Figure A in S2 File). We bootstrapped the correlation coefficients 50 times to estimate their confidence intervals. For this analysis, we used the package “pse” for R [32].

### Two-species model exploration

We used the model to explore how population persistence, parasitism rates and the proportion of resistant hosts responded to parameters related to the additive genetic variance of the weevil hosts and parasitoids. We studied the models via numerical simulations, given the difficulty of deriving interpretable analytical expressions for the dynamic of the systems [21]. The simulation started with the parasitoid at low densities (10 m^-2^) and the host at ecosystem carrying capacity. We studied how the output of the models changed as a function of the parameters shown to most strongly influence the results of the model via the sensitivity analysis. We performed this analysis within the empirically-derived parameter spaces described in Table 1.

### Comparison with field data

To assess the plausibility of our model predictions, we compared the model results with field data. We ran the two-species model for both systems, *L*. *bonariensis* with *M*. *hyperodae* and *S*. *discoideus* with *M*. *aethiopoides*, for the range of parameters described above under the heading “model parameterization and study cases”. When only one measured value was available for a particular parameter, we used only that value in the model, otherwise we sampled 20 values from inside the range of values found for a given parameter in the literature (see Table 1). The survival of parasitoid larvae (c) was only available for one of the parasitoids; therefore we assumed the same value for both species. It is important to note that the model parametrization was totally independent of the model results; i.e. the studies of field parasitism rates were not used to parameterize the models.

For this field versus model comparison, we used the field data on nationwide parasitism in *L*. *bonariensis* with *M*. *hyperodae* published by Tomasetto et al. (2017 [12]). We compared the parasitism rate values at the beginning of the biocontrol programme (three years after the introduction of the parasitoid) and those available from the latest field data (24 years after the introduction of the parasitoid) with model data obtained over the same time frame: 9 and 72 generations, because *L*. *bonariensis* has a maximum of three generations per year [33].

For the model validation in the *S*. *discoideus* with *M*. *aethiopoides* system, we used field data from two sources: three years after the introduction of the parasitoid, from Goldson et al. (1990 [34]); and 12 years after the introduction of the parasitoid, from Kean and Barlow (2000 [35]). We compared these data with parasitism rate obtained from the model simulations for the same time frame: 3 and 12 generations, because *S*. *discoideus* has only one generation per year.

Although it could be argued that 1–3 generations per year are few for studying evolution, rapid evolution of resistance to sexually [36] and asexually [12]) reproducing parasitoids has been reported in the wild, and rapid (within few seasons) evolution of resistance to pesticides in insects is well known (e.g. Sudo et al. 2018 [37]). Thus, the timescales of this comparison are congruent with those at which insect evolution is known to occur. For both host-parasitoid pairs, we used a simple two sample Student’s t-test to determine if the model data (mean across all runs, with a range of parameter values described above) and the field data (mean across different sites and dates for the same year) differed significantly for the two time steps chosen. We tested the data for normality and we performed an F-test to compare the variances of the different data sets. If the variances differed significantly, we estimated them separately for each group and used the Welch modification to the degrees of freedom in the t-test. We also calculated for both systems the predicted parasitism rates at 100 years after the introduction of the parasitoids.

### Extension of the model framework (“three-species model)

We then extended the “two-species model”, as described above, to generate a model where three species interact with each other, in the form of two parasitoids and one host (hereafter: “three-species model”). This three-species model is a modification of the original model by Hochberg and Holt (1995), which allowed us to study the consequences of introducing a new parasitoid after an existing parasitoid has already become established in the system. The basic system of equations for this three-species model is as follows:

$$N_{t+1}=\lambda \;N_{t}g\left\{N_{t}\right\}\;\left(\alpha_{p}+\left(1‑\alpha_{p}\right)f\left\{N_{t},P_{t}\right\}\right)\left(\alpha_{w}+\left(1‑\alpha_{y}\right)h\left\{N_{t},Y_{t}\right\}\right)$$ (9)

$$P_{t+1}=c_{p}N_{t}g\left\{N_{t}\right\}\left(1‑\alpha_{p}\right)\left(1‑f\left\{N_{t},P_{t}\right\}\right)h\left\{N_{t},Y_{t}\right\}$$ (10)

$$Y_{t+1}=c_{y}N_{t}g\left\{N_{t}\right\}\;\left(1‑\alpha_{y}\right)\left(1‑h\left\{N_{t},Y_{t}\right\}\right)f\left\{N_{t},P_{t}\right\}$$ (11)

The new parasitoid, Y, is introduced to the system after the first host-parasitoid (N-P) system had undergone 500 generations since the onset of the simulation (preliminary exploration suggested that this was enough for the first host-parasitoid system to stabilise). Varying the number of generations before the introduction of the second parasitoid did not change the overall result of the model. When the second parasitoid is introduced, the parasitism rate it exerts is determined by the character values of the existing host (bearing in mind that this character value had been changing due to coevolution with the first parasitoid), and the character value of the new parasitoid (which has the same value as that which the first parasitoid had when introduced). The parameters for this system of equations are essentially the same as those described above for the “two-species model”, however note that the parasitoid parameters are specific to each parasitoid and are denoted by the subscript “p” and “y”, for the first and second parasitoid respectively. Likewise, *h*{N_t_, Y_t_} represents the proportion of weevils unparasitised by the added second parasitoid. For this model we assumed that there is not a competitive hierarchy between the two parasitoids, and that parasitoids of one species cannot parasitise a host that has already been parasitised by the other species. Both parasitoids attack at the same time and the proportion of weevils available to be parasitised by each parasitoid depends on the proportion parasitised by the other parasitoid and the proportion of the host population that is resistant to each parasitoid. The host resistance level is not totally independent for each parasitoid in the “three-species model”. The model assumes that resistance depends on a single character in each species, designated as n for the weevil and p and y for the two parasitoids; the character n that governs host resistance is the same for both parasitoids. The proportion of weevils resistant to parasitism is defined as a function of the average character value of both species:

$$\alpha_{pt}\left\{\bar{n},\bar{p}\right\}=1‑exp\left\{‑{\left({\bar{n}}_{t}-{\bar{p}}_{t}\right)}^{2}\right\}$$ (5A)

$$\alpha_{yt}\left\{\bar{n},\bar{y}\right\}=1‑exp\left\{‑{\left({\bar{n}}_{t}-{\bar{y}}_{t}\right)}^{2}\right\}$$ (5B)

The changes in the average value of the character of the host is therefore driven by its change in fitness, which is the result of the interaction with both parasitoids.

$${\bar{n}}_{t+1}={\bar{n}}_{t}+\Gamma_{n}\left[\partial ln\;W_{n}/\partial \bar{n}\right]$$ (6A)

$${\bar{p}}_{t+1}={\bar{p}}_{t}+\Gamma_{p}\left[\partial ln\;W_{p}/\partial \bar{p}\right]$$ (6B)

$${\bar{y}}_{t+1}={\bar{y}}_{t}+\Gamma_{y}[\partial ln\;W_{y}/\partial \bar{y}]$$ (6C)

$$W_{n}=N_{t+1}/N_{t},\;W_{p}=P_{t+1}/P_{t}\;and\;W_{y}=Y_{t+1}/Y_{t}$$

For the purposes of starting research on what would be the consequences of introducing populations of parasitoids with different reproductive strategies and to keep the model simple, we assumed that both populations of parasitoids are exactly the same except for their additive genetic variance (Γ, as a proxy for their reproductive strategy) and their intrinsic attack rate (η). This would be possible if strains of the same species of parasitoid, but with different reproductive strategies, were introduced. This is a strong assumption of the three species model, and we take it into account when discussing the results of the simulations. For this model we took the extreme case of assuming that the asexual parasitoid had 0 additive genetic variance, but this was balanced by having double the intrinsic attack rate (η) of the sexual parasitoid with additive genetic variance greater than 0. This double attack rate of the asexual line represents a case where the sexual parasitoid has a 1:1 sex ratio, so only half of its population (the females) produce offspring. A description of the model extension to the three species case and the R [38] script used for its implementation are presented in S1 File.

## Results

### Two-species model and sensitivity analysis

Even though most of the relationships between the model output used for the sensitivity analysis (the proportion of resistant hosts) and the parameters tested were significant (Table 2), only four parameters each explained 20% or more of the variation: additive genetic variance of the host and that of the parasitoid, the spatial heterogeneity of parasitism in the field and the intrinsic growth rate of the host. The two most important parameters determining the evolution of resistance were the additive genetic variance of the host and that of the parasitoid, which represent the abilities of each population to respond to the selective pressure of the other. We used the additive genetic variance as a proxy for the reproductive strategy of each species. At the extreme, an asexual population would have 0 additive genetic variance, assuming that beneficial mutations would be negligible in comparison with genetic recombination by sexual reproduction. The partial rank correlation coefficients between the additive genetic variance of the host and parasitoid, and the proportion of resistant hosts were 0.73 and -0.71 respectively, explaining most of the variation in resistance (Table 2).

**Table 2:** Results for the sensitivity analysis of the “two-species model” for 6000 simulations.

Parameter	Definition	Partial rank correlation coefficient	p-value
г_n_	Additive genetic variance of host	0.73	*< 0*.*0001*
г_p_	Additive genetic variance of parasitoid	-0.71	*< 0*.*0001*
κ	Spatial heterogeneity in parasitism	0.49	*< 0*.*0001*
λ	Intrinsic growth rate of the host	-0.20	*< 0*.*0001*
K	Host carrying capacity (adults/m^2^)	0.16	*< 0*.*0001*
p_0_	Initial parasitoid character	0.11	*< 0*.*0001*
C_n_	Cost to host character	0.10	*< 0*.*0001*
n_0_	Initial host character	-0.09	*< 0*.*0001*
c	Survival of parasitoid larvae	0.09	*< 0*.*0001*
η	Intrinsic rate of attack of the parasitoid	0.05	*< 0*.*0001*
N_0_	Initial host density	0.02	0.063
P_0_	Initial parasitoid density	0.01	0.248
C_p_	Cost to parasitoid character	0.001	0.909

The partial rank correlations are between the proportion of resistant hosts (result from the model) and each parameter.

The spatial heterogeneity of parasitism in the field and the intrinsic growth rate of the host were the next most important parameters. The partial rank correlation coefficients between these parameters and the proportion of resistant hosts were 0.49 and -0.20 respectively (Table 2). Thus spatial heterogeneity in parasitism (κ) is a measure of parasitism aggregation. As the value of κ increases, the heterogeneity in parasitism decreases. Here small values of κ indicate aggregation in parasitism while higher κ values approach a more random distribution. The positive correlation between the spatial heterogeneity and host resistance shows that aggregation in parasitism is an important influence on resistance, which decreases as parasitism aggregates. The negative correlation coefficient between the intrinsic growth rate of the host and its resistance is not surprising, as it matches the model prediction in the original paper by Hochberg and Holt (1995 [21]). They showed that for lambda values of 12 or higher, the system is pushed towards a bottom up control, where both populations and their refuge characters evolve and equilibrate to constant levels, with the parasitoid only moderately suppressing the host. This happens only when parasitoids and hosts have close to, or equal levels of additive genetic variance (meaning in our model that both reproduce sexually). This is because the host is able to reproduce disproportionately faster than the parasitoid (resulting in higher fitness and faster changes in the host character value. This creates an imbalance where parasitoid numbers decrease until they stabilise with very low values for the number of host resistant to parasitism. This can be seen in Figure C in S2 File, where the bottom two lines show the change over time in resistance for lambda values of 20 and 40. It is also interesting to note that *C*_*p*_, which represents the cost for the parasitoid to evolve, was not significantly correlated with the proportion of resistant hosts. Also, the initial population sizes of the parasitoid and host were not significantly correlated with the proportion of resistant hosts of the model (Table 2).

**Fig 1:**
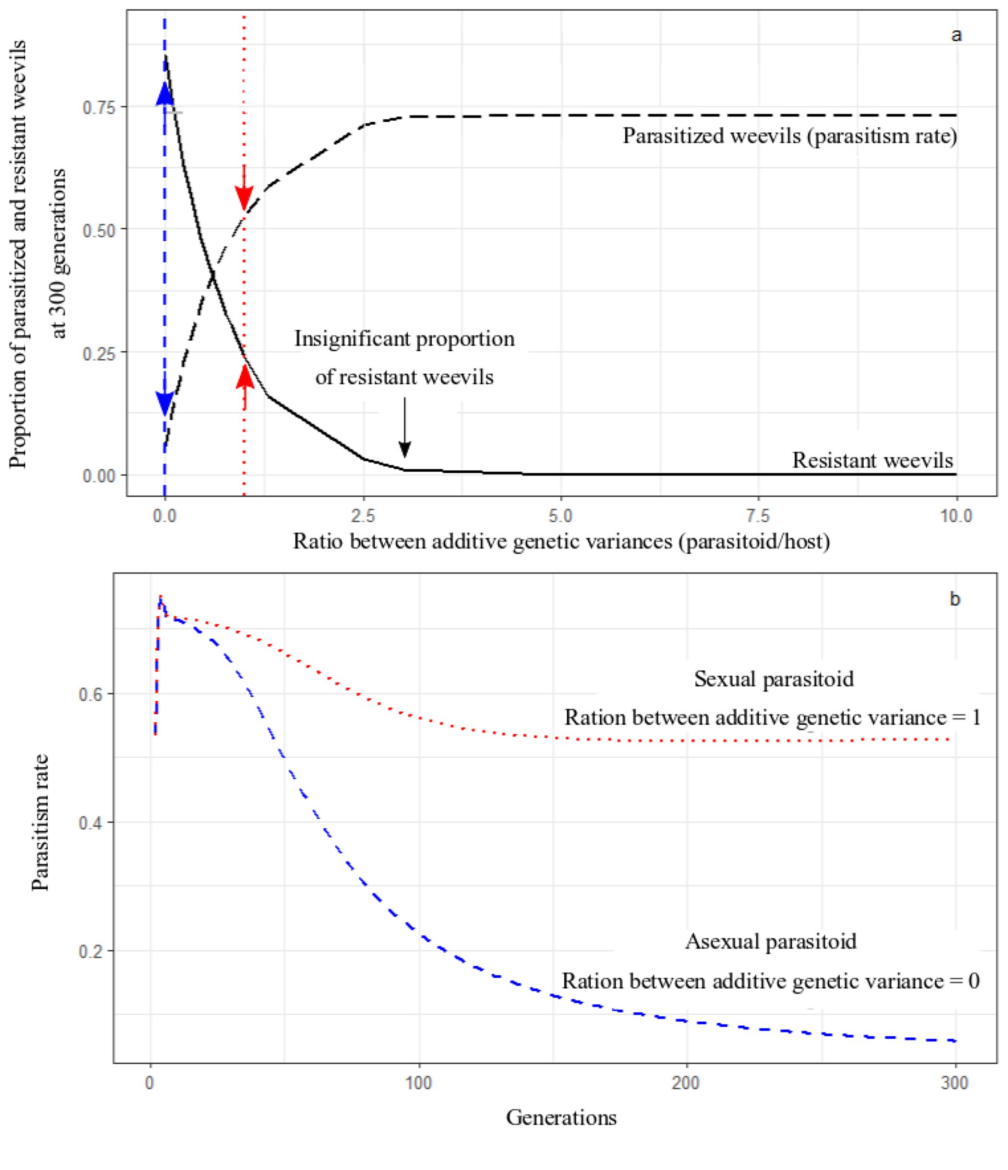
Effect of additive genetic variance on parasitism rates and resistance. a) Proportion of parasitized and resistant weevils at 300 generations for different ratios between the additive genetic variance (AGV) of the parasitoid and the host. All parameters were kept constant, except for the parasitoid AGV. The dashed blue line and the blue arrows show resistance and parasitism rate of an **asexual** parasitoid interacting with a **sexual** host (parasitoid AGV = 0 and host AGV = 0.1). The dotted red line and the red arrows show resistance and parasitism rate of a **sexual** parasitoid interacting with a **sexual** host (parasitoid AGV = 0.1 and host AGV = 0.1). b) Examples of the parasitism rates over generation time for an **asexual** parasitoid (blue dashed line) and a **sexual** parasitoid (red dotted line).

Further analysis of these parameters showed that the ratio between the additive genetic variance of the parasitoid and the host, Γ_p_: Γ_n,_ alone determines the general patterns of parasitism rates and the evolution of resistance in the host (Fig 1A). A lower ratio between parasitoid and host additive genetic variance leads to a higher proportion of hosts evolving resistance and consequently lower parasitism rates over time (from a parasitism rate of 5% when the parasitoid had 0 additive genetic variance to a parasitism rate of 72% when the parasitoid had more than 0.03 additive genetic variance; the host additive genetic variance was held constant at 0.01). Moreover, the additive genetic variance of a given parasitoid must be at least three times higher than that of the host to prevent the emergence of resistance in the host population (Fig 1A). An example of a comparison between an asexual parasitoid (with no additive genetic variance) and a sexual parasitoid (with additive genetic variance equal to the host) is given in Fig 1B. Varying other parameters such as the growth rate of the host or the spatial heterogeneity of parasitism did not qualitatively change the patterns described above (S2 File).

### Comparison with field data

**Fig 2:**
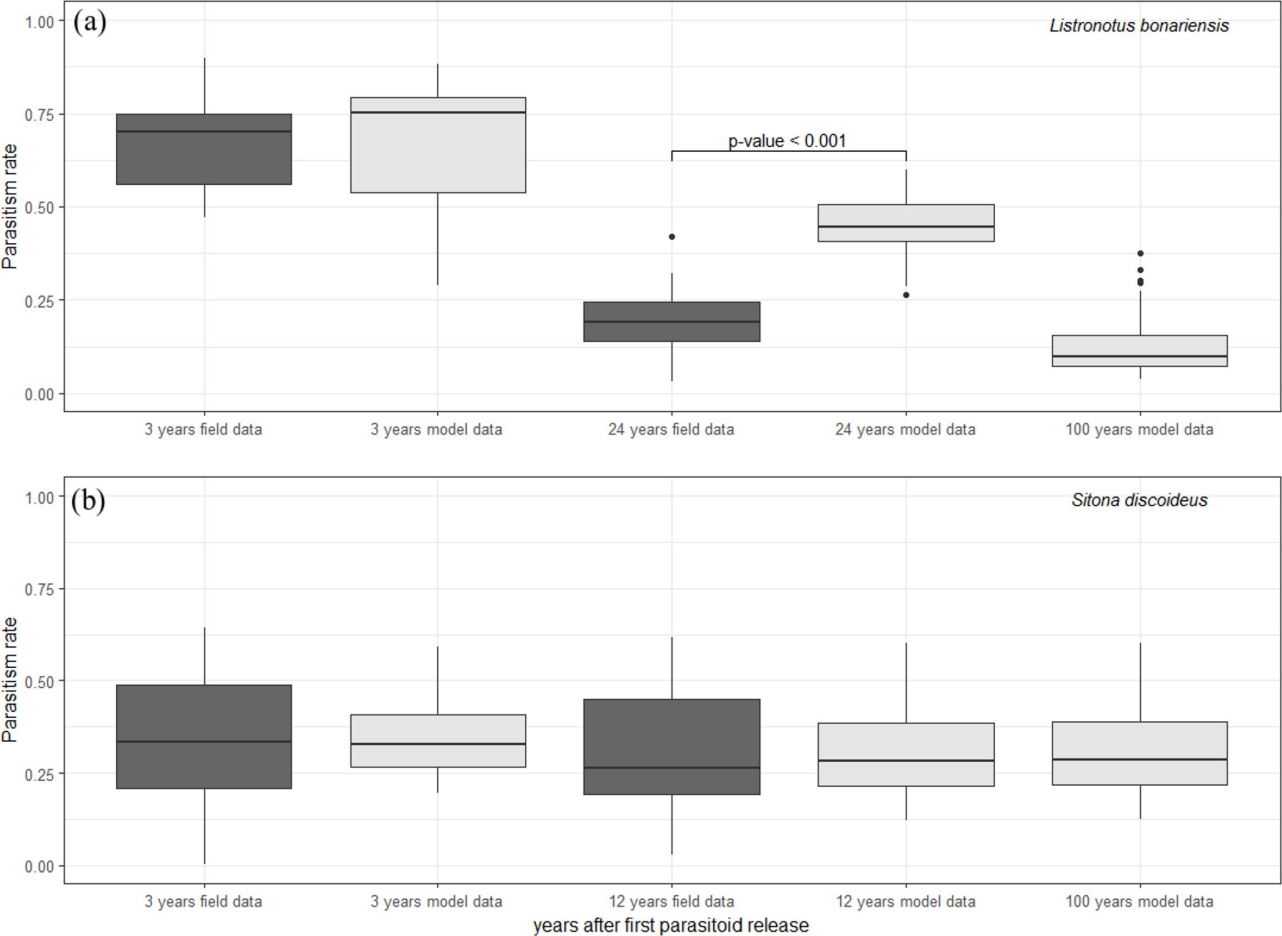
Comparison between the field data and the model results for both systems. a) *L*. *bonariensis* and *M*. *hyperodae*; b) *S*. *discoideus* and *M*. *aethiopoides* (Moroccan strain). The upper and lower "hinges" represent the first and third quartiles. The whiskers extend from the hinge to the highest and lowest value that is within 1.5 * distance between the first and third quartiles. The points beyond the end of the whiskers are outliers.

The two-species model for *L*. *bonariensis* and *M*. *hyperodae* predicted a decline in parasitism rates over time, which matched the field data at the start of the biocontrol implementation (three years after the introduction of the parasitoid, p-value = 0.85, Fig 2A). This model also predicted the magnitude of the decline, but over a much longer time scale, such that it predicted higher parasitism rates than were present in the field for the last year of available field data (24 years after the introduction of the parasitoid, p-value = 0.001, Fig 2A). The model predicted an ongoing decrease in parasitism rate for 100 years after the introduction of the parasitoid, culminating with an average parasitism rate of 0.12 (7% lower than the most recent field data and 32% below the model predictions for 24 years after parasitoid introduction, Fig 2A).

For *S*. *discoideus* and *M*. *aethiopoides* the two-species model results were not significantly different from the field data, and showed that the parasitism rates in this system would not change over time (p-value = 0.45 for the 3^rd^ year after the introduction of the parasitoid; p-value = 0.92 for the 12^th^ year after the introduction of the parasitoid). Further, the model predicts that they will remain at these levels 100 years after release (Fig 2B). Of course, correlations between model predictions and field observations are not evidence of any mechanism. However, this congruence demonstrates that our model parameterisation generates predictions that fall within the realms of biological reality.

### Three-species model

**Fig 3:**
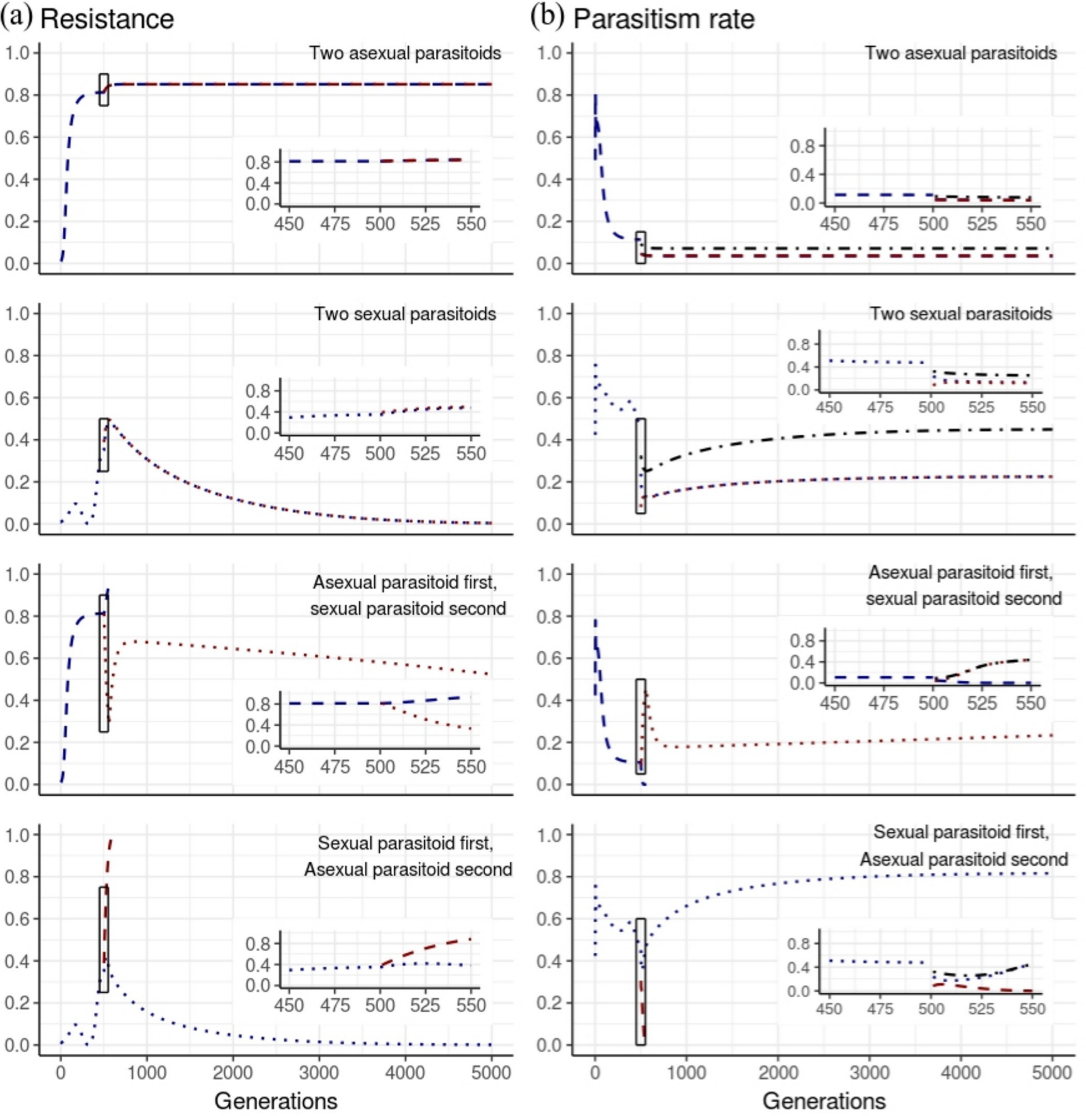
Four examples of parasitism rates and resistance over generation time for the three species model (two parasitoids and one host). The y axis shows parasitism rate (a) and proportion of resistance (b). The blue line represents the parasitoid that was introduced first, and the red line represents the parasitoid introduced second (at 500 generations). The dashed line represents an asexual parasitoid, and the dotted line represents a sexual parasitoid. The dash-dotted line represents the sum of the parasitism rate of the two parasitoids. The inset graphs are expansions of the box where the second parasitoid was introduced (showing 50 generations before and after the introduction). Parasitoids are extinct when the parasitism rate equals 0 and the resistant proportion equals 1.

The three-species model showed that a sexual parasitoid surpasses the parasitism rate of an asexual parasitoid over time, and eventually drives the asexual parasitoid to extinction, irrespective of the order in which the two variants were added. The fate of a second parasitoid that is introduced into a system where there already is a parasitoid interacting with the same host depends on its additive genetic variance (Table 3, Fig 3). The only instance where an asexual parasitoid survives the introduction of another parasitoid is when another asexual parasitoid is also introduced to the system. However, in this case, the parasitism rate exerted by both of these parasitoids is lower than the parasitism rate of the first parasitoid alone. In an asexual parasitoid system where the populations are at equilibrium (albeit with a very low parasitism rate), the introduction of a new parasitoid perturbs that equilibrium and, in all cases studied, further lowers the parasitism rate of the first parasitoid. In a sexual parasitoid system, the new asexual parasitoid does not persist and its parasitism levels decline at a rate higher than if this parasitoid were alone. Varying the growth rate of the host or the spatial heterogeneity of parasitism of either parasitoid does not qualitatively change any of the patterns of results described above (S2 File).

**Table 3:** Parasitism rates resulting from different combinations of additive genetic variance of parasitoids for the three species model.

Additive genetic variance of the first parasitoid	Additive genetic variance of the second parasitoid	Parasitism rates before the introduction of the second parasitoid	Parasitism rates at the end of simulation (first parasitoid–second parasitoid)
0	0	0.06	0.03–0.03
0	0.01	0.06	**Extinct**– 0.50
0	0.1	0.06	**Extinct–** 0.65
0.01	0	0.4	0.84 –**Extinct**
0.01	0.01	0.4	0.22–0.22
0.01	0.1	0.4	0.23–0.23
0.1	0	0.5	0.82 –**Extinct**
0.1	0.01	0.5	0.23–0.23
0.1	0.1	0.5	0.24–0.24

Parasitism rates are calculated as the proportion of all hosts that are infected by a given parasitoid, so the total proportion of hosts infected is the sum of the two attack rates.

These results (Table 3, Fig 3 and S2 File) show that, if there is at least one sexual parasitoid in a given system, greater diversity of parasitoids reduced the proportion of resistant hosts in that system. Even when the asexual parasitoids go extinct after being introduced with a sexual competitor; the resulting parasitism rate of the sexual parasitoid remains higher than it was prior to the introduction of the asexual parasitoid. This elevated parasitism rate occurs because the asexual parasitoid promotes more rapid evolution of the host, and the rapid change in character values of the sexual parasitoid to compensate for this rapidly-evolving host results in higher parasitism rates after the asexual parasitoid goes extinct. This applies even if it had never existed in the system. Overall, diversity of asexual parasitoids did not improve the overall levels of parasitism and even allowed the host population to increase its resistance levels relative to when a single parasitoid species was present.

Because the character that governs resistance in the weevil is the same for both parasitoids, when both parasitoids had the same additive genetic variance, upon the introduction of the second parasitoid, the host population automatically has a higher resistance rate to the new parasitoid than to the parasitoid to which it has been exposed for a long time, and with which it has co-evolved. The new parasitoid has to “catch up” by evolving its character value to be able to parasitise the weevil at the same rate as the older parasitoid in the system. This assumption also explains why the parasitism rate in a system with two asexual parasitoids becomes lower than in a system with just one and why parasitism rate of an asexual parasitoid decreases more rapidly when it is in a system that also includes a sexual parasitoid.

## Discussion

Our model revealed that, unless parasitoids have at least three times the genetic variance of their hosts, the evolution of some level of host resistance is inevitable in the long term. Although the dependence of evolution on additive genetic variance is not surprising (and in fact is described by the well-known breeder’s equation [39]), there are two key aspects of our findings in this contribution. The first is that the additive genetic variance of parasitoids must be disproportionately greater than that of the host to avoid evolution of resistance. Second, the relationship between the ratio of parasitoid-host additive genetic variance and the emergence of resistance is not linear. In the case of a sexual parasitoid, such resistance is relatively minor, and stabilises sooner, whereas asexual parasitoids invoke substantial host resistance and a long-term decline to very low parasitism rates (Fig 1B). That parasitoids with greater genetic variance are more successful in co-evolutionary arms races has been shown with a pure co-evolutionary model approach before (not including a population dynamics component [40]). This latter scenario reflects the recently reported 44% decline in mean parasitism rates over two decades observed in the Argentine stem weevil [12]. The generally low levels of parasitism found in the native range of this pest by *M*. *hyperodae* (average of 15.8% [41]) also coincide with the long-term predicted attack rate of our model. However, resistance of the Argentine stem weevil to its parasitoid in the field evolved more quickly than our model predicted (Fig 2A). One possible hypothesis is that we underestimated the additive genetic variance in the weevil host. Further, the results from our three-species model, where an asexual parasitoid is introduced to a system already containing a sexual parasitoid, gives us another possible hypothesis to explain the difference between the model results and the field data. In this model case, parasitism rates by the asexual parasitoid decline faster than when it is interacting with the host alone. To this effect, Barratt et al. (2007 [42]) have shown that *L*. *bonariensis* is also parasitized by the sexual parasitoid *M*. *aethiopoides* at very low levels. Thus, it is possible that this accelerated the decline in parasitism rates by *M*. *hyperodae*. However, more research is needed to study and test this hypothesis fully.

Our results on the importance of the reproductive strategy in biological control are supported by the work of Burdon and Marshall (1981 [43]) on the reproductive strategy of weeds and their biological control. They found that sexually reproducing weeds were more difficult to control than their asexually reproducing counterparts. This scenario is illustrated in Fig1A, where we can substitute the ratio of additive genetic variance between parasitoid and host by the ratio of additive genetic variance between the herbivore and its weed host. Here we see that when the additive genetic variance of the weed gets disproportionally smaller than the herbivore (as in a situation where the herbivore reproduces sexually and the weed asexually), the weed would not evolve resistance to the herbivore.

Our model results suggest that even when both species in a parasitoid-host system reproduce sexually, resistance can in fact still evolve depending on the ratio between the additive genetic variance of both species. In a thorough laboratory experiment looking at the space-time component of the population dynamics of house flies and their parasitoid, *Nasonia vitripennis*, Pimentel et al. 1963 [44] showed that the reproductive capacity of the parasitoid declined by between 40% and 68% within 8 and 20 generations respectively. These results agree with our model results when both species have some degree of additive genetic variance; with additive genetic variance ratios varying between 0.5 and 10, there is a decrease of between 44% and 1% in parasitism rates respectively from the start of the simulation to 300 generations (Fig 1A). This also relates to our results regarding the New Zealand parasitism rates of the lucerne weevil by its sexually reproducing parasitoid, where parasitism rates were found to be ~25–50% (year average). In Australia, where the lucerne weevil was also introduced and is considered a pest, the parasitism by *M*. *aethiopoides* was found to be less than 25% [45]. The parasitism levels in this weevil’s and its parasitoid’s native range of Morocco are very low, averaging less than 10% of parasitised weevils among different regions [45]. However, these low levels of parasitism by *M*. *aethiopoides* in its native range might be caused by the complexity of the ecosystem and interfering species that are absent in the New Zealand pastures.

One of the best-known cases of biological control success is the use of the asexual parasitoid *Encarsia formosa* for the control of whiteflies in greenhouses. Two biological control strategies are successfully used for this system: an inundative release method, where *E*. *formosa* is released periodically in large numbers to obtain an immediate control effect; and a seasonal inoculative release method, where *E*. *formosa* is released in large numbers in short-term crops to obtain an immediate control effect as well as a build-up of the *E*. *formosa* population for control later during the same season [46]. The nature of the strategies used in this system will prevent the evolution of resistance in the hosts, as the host population from one season to the next is completely different. Moreover, many aphid species reproduce asexually for at least part of their life cycle, which eliminates genetic recombination [47] and places the evolutionary asymmetry in favour of the parasitoid.

The importance of additive genetic variance suggests that, for effective long-term control of a given pest, the genetic variability of the introduced control agent should be maximized. This idea has been noted by several authors in the past [5,48–50]. Here we showed that the additive genetic variance of a control agent has to be disproportionately greater than that of the host for achieving a successful biocontrol program. Avoiding severely bottlenecked populations of the control agent is as important as trying to introduce a control agent that has the same or even greater additive genetic variance (via its reproductive strategy) than its target species. Moreover, we can extrapolate from the model results that it may be more challenging to control pests that have been established for a long time (so have accumulated more variability by mutation and recombination), those that have been introduced multiple times on the same region, or native pests.

It has been suggested that a possibility for improving the long-term success of biological control using parasitoids would be to first introduce an asexual form of the parasitoid, followed by additional later releases of a sexual form [18]. The results of our “three-species model” supported this idea showing that a sexual form of the parasitoid that is introduced after an asexual form has been established will succeed and out-compete the asexual form. These results are important when the success of an asexual form of a parasitoid is already declining, as in the case of *M*. *hyperodae*, and the introduction of a sexual parasitoid could improve the control of the host: in this case the Argentine stem weevil.

Our model results showed that asexual parasitoids do not persist if they have to compete with a sexual parasitoid of similar characteristics. Sexual and asexual parasitoids of the same or different species that attack the same host are known to co-exist in nature (e.g. *Lysiphlebus fabarum* [51]; *Trichogramma spp*. [52]; *Venturia canescens* [53]). Moreover, it is possible that many cases of coexisting sexual and asexual strains of parasitoids have been overlooked, and this scenario might be more common than is known from the literature. However, there are several examples where the co-existence of both reproductive forms could be explained by a geographic or niche segregation (see Mitsui and Kimura 2010 [54] for some examples in the islands of Japan, and Amat et al. 2006 [55] for an example on the sexual and asexual forms of the species *Venturia canescens*). Also, work on sexual and asexual populations of *Lysiphlebus fabarum* in Europe showed that it is possible that, in some cases, the groups of sexual and asexual populations of a species of parasitoid represent a young complex of lineages that are not completely isolated between reproductive modes [56]. The lack of genetic isolation between these populations would explain their coexistence.

An assumption of the “three-species model” is that the character that governs resistance in the weevil population is the same for both parasitoids. We made this assumption because many haplodiploid Hymenoptera are capable of both sexual and asexual reproduction. In such systems, it is probable that host resistance to different parasitoid individuals is not influenced by sexual reproduction. Also making this assumption, Ikegawa et al. (2014 [57]) investigated the effects of predator-nonspecific adaptive defences on the success of biological control using a theoretical modelling approach. Our results matched their findings; predator–nonspecific defences enhanced three species coexistence (in the case of all having the same ability to evolve), but the introduction of two natural enemies rarely improved the efficiency of biological control. This assumption is also supported by empirical evidence of several cases where defences against natural enemies are nonspecific, from physical and behavioural defences to generalized immune responses. Several examples of the first two types are found in a review on insect behaviour and morphological defences against parasitoids by Gross (1993 [58]). There is also evidence that immune response to parasitoid infection can be non-specific, as shown by Fellowes et al. (1999 [59]) on encapsulation of three different species of parasitoid larvae by *Drosophila melanogaster*. Furthermore, our results showed that after the introduction of a second parasitoid, the host population immediately has a higher resistance rate to the new parasitoid. Our results in conjunction with the work of Ikegawa et al. (2014 [57]) and the above evidence suggest that whether defence traits of pests are non-specific may be important in determining the potential success or failure of a “multiple enemies” approach for biological control of pests.

Overall, our findings illustrate that the asymmetry in the reproductive strategies between parasitoids and hosts (and the extent of differences in additive genetic variance between them) is crucial for predicting their long-term success as biological control agents. Long-term monitoring of biological control efficacy is rare, and resistance may be more common than known examples would imply [12]. In general our findings suggest that the use of parasitoids with lower additive genetic variance than their host (due to asexuality or severe population bottlenecks) is likely to promote resistance, unless there are repeated reintroductions to prevent evolutionary dynamics. Fortunately, introduction of sexually reproducing individuals after an initial introduction of asexual strains may overcome these problems. Moreover, our results would also suggest that biological control of an asexual pest (e.g. some aphids) or pests that have recently colonized following an extreme bottleneck will be more sustainable. Irrespective, we suggest that evolution must be considered when evaluating the long-term risks and benefits of classical biological control.

## Supporting information

S1 File Model development, R code and information about parameters values. (PDF)

S2 File Results from the sensitivity analyses and figures illustrating the results from the “two species model” and the “three species model”. (PDF)
