# Supplementary material for: Asymmetry in reproduction strategies drives evolution of resistance in biological control systems
Source: PLoS One. 2018 Dec 19;13(12):e0207610. doi: 10.1371/journal.pone.0207610 (PMC6300327; doi:10.1371/journal.pone.0207610)
Supplement: S1 File — (PDF) [file pone.0207610.s001.pdf]

S1 File. Model development, R code and information about parameters values.

Co-Evolutionary model adapted from Hochberg and Holt (1995).

First we describe the “two-species model” incorporating the script of the model. The model was writen in R [1], and the code is inbeded in the description.

For coding propurses, we need to define a function that will contain the model procedure. The function is called `"CoEvoModel_Two_table"` and all the parameters of the model are defined in between the parenthesis after `"function"`.

```
CoEvoModel_Two_table <-
function(Lambda,K,n_p,c_p,k_p,a_p,N_0,P_0,AgH,AgP,C_n,C_p,s,n_0,p_0)
{
```

Here we set the maximum generation time (gtime) that the simulation will run for:

```
gtime <- 50000
```

We need to make a table to save the results of the model, with a column for each parameter that changes over time: H_density = density of the host, P_density = density of the parasitoid, H_character = value of the host character, P_character = value of the parasitoid character, Proportion_P = the proportion of host attacked (of the total population of hosts not including the ones in the refuge), Parasitism_rate_P = parasitism rate, alpha_value_P = proportion of host not available to the parasitoid (in a refuge).

```
Results_table <- data.frame(timestep= numeric(gtime), H_density= numeric(gtime),
P_density= numeric(gtime), H_character= numeric(gtime), P_character=
numeric(gtime), Proportion_P= numeric(gtime), Parasitism_rate_P= numeric(gtime),
alpha_value_P= numeric(gtime))

Results_table[Results_table == 0] <- as.integer("")

Results_table$timestep <- c(1:gtime)
```

Initial values for density and character for the parasitoid and host are introduced at this point:

```
#Initial value for host density
Results_table$H_density[1] <- N_0

#Initial value for the first parasitoid density
Results_table$P_density[1] <- P_0

#Initial value for the host character
Results_table$H_character[1] <- n_0

#Initial value for the parasitoid character
#(host character >= parasitoid character is a constraint of the model)
```

```
Results_table$P_character[1] <- p_0
```

The model runs in a loop, where each step represents one generation (from 1 to gtime). In each step, we update the parameters that change over time, the density of host and parasitoid, and the parasitism rate.

```
#Here starts the loop
for(t in 1:(gtime-1)) {
```

Here we define the function for the proportion of host not available for the parasitoid (in a refuge) in generation t, equation 5a on the main body of the paper:

$$\alpha_t\{\bar{n}, \bar{p}\} = 1 - \exp\{-(\bar{n}_t - \bar{p}_t)^2\} \quad (5a)$$

```
alpha_f <- function(n,x){1 – exp(-s*(n-x)^2)}
```

Here we define the function for the host realized intrinsic growth rate, which takes into account the cost of changing the character that provides resistance (equation 7 in the main body of the paper):

$$\lambda_t\{\bar{n}_t\} = \lambda^* - C_n\bar{n}_t \quad (7)$$

```
#Function for the host realized intrinsic growth rate in generation t
#This Lambda is not included in the density dependent survival of equetion
#g(N_t)

Lambda_f <- function(n){Lambda – C_n*n}
```

Here we define the function for the intrinsic attack rate for the parasitoid, which takes into account the cost of changing the character that provides "virulence" (equation 8 in the main body of the paper):

$$\eta_t\{\bar{p}_t\} = \eta^* - C_n\,\bar{p}_t \quad (8)$$

```
#Function for the realized intrinsic attack rate for the parasitoid
#Here x is the character value in gtime t for a given parasitoid
#n_x is the original attack rate for the parasitoid (n_p)
Attack_f <- function(n_x,x)(n_x – C_p*x)
```

To start the calculations for this generation, we need to read the values for the densities and character values from this gtime step:

```
N_t <- Results_table$H_density[t]
P_t <- Results_table$P_density[t]
n_t <- Results_table$H_character[t]
p_t <- Results_table$P_character[t]
```

We calculate the realized growth rate for the host inside of the refuge (equation 7):

```
LambdaM <- Lambda_f(n_t)
```

And calculate the realized attack rate for the parasitoid (equation 8):

```
n_p_M <- Attack_f(n_p,p_t)
```

Here we calculate the proportion of host not available to be parasitized (in a refuge, equation 5a):

```
alphaM <- alpha_f(n_t,p_t)
```

Here we write the function for equation 3, describing the proportion of host escaping density dependent mortality, this function will be used to calculate this proportion in each step:

$$g\{N_t\} = [1 + N_t (\lambda - 1)/K]^{-1} \qquad (3)$$

```
g<-function(N){(1 + N * (Lambda – 1)/K)^-1}
```

And here we write the function for equation 4, describing the proportion of host OUTSIDE the resistant group (the refuge) that survive parasitism:

$$f\{N_t, P_t\} = [1 + (a\, P_t / k(1 + a\, N_t\, g\{N_t\}(1 - \alpha)/\eta]^{-k} \qquad (4)$$

```
#Note that the cost to the attack rate of the parasitoid is included in the
equation already
#= n_p – C_p*p_t

f<-function(N,P){(1 + (a_p * P)/(k_p * (1 + a_p * N * g(N) * (1 - alphaM)/(n_p-
C_p*p_t))))^-k_p}
```

Here we will calculate the densities of host and parasitoid, as well as the changes in character, the proportion of host in the refuge (resistant) and the parasitism rate for this time step t

Using equation 1 we calculate the density of host at time t + 1

$$N_{t+1} = \lambda\, N_t\, g\{N_t\}\ (\alpha + (1\text{-}\ \alpha)\, f\{N_t, P_t\}) \qquad (1)$$

Note that the co-evolutionary part of the model is included in `f(N_t,P_t)`, in `LambdaM` and in `alphaM.`

```
#Density of host at gtime t+1
N_t1 <- LambdaM * N_t * g(N_t) * (alphaM + (1-alphaM) * f(N_t,P_t))
```

Using equation 2 we calculate the density of parasitoids at time t + 1

$$P_{t+1} = c\, N_t\, g\{N_t\}\ (1\text{-}\ \alpha)\ (1 - f\{N_t, P_t\}) \qquad (2)$$

```
#Density of parasitoid at t+1
P_t1 <- c_p * N_t * g(N_t) * (1-alphaM) * (1-f(N_t,P_t))
```

Here we calculate the changes in phenotipic means to then calculate the changes in the value of the character. For doing so, we calculate the derivative of the natural log of the changes in densities from time t to time t + 1 with respect to the changes on the character value from time t to time t +1. See Lande (1975) and Lande and Arnold (1983) for an explanation for this processes.

```
#Evolution of phenotypic means
# d ln (N_t1/N_t) / d n_t -> 1/(N_t1/N_t) * d (N_t1/N_t) / d n_t
# the change on the fitness of the host / the change on the host character

eq1_D <- function (n_deriv)
{
.e1 <- n_deriv - p_t
.e2 <- exp(-(s * .e1^2))
.e3 <- 1 + N_t * (Lambda - 1)/K
.e5 <- n_p - C_p * p_t
.e7 <- 1 + a_p * P_t * (1 + a_p * N_t * .e2/(.e3 * .e5))/k_p
.e9 <- 1/.e7^k_p - 1
(s * (2 * (a_p^2 * N_t * P_t * .e2/(.e7^(1 + k_p) * .e3 *
.e5)) - 2 * .e9) * .e2 * (Lambda - C_n * n_deriv) * .e1 -
C_n * (1 + .e9 * .e2))/.e3
}
```

After formulating the above function for the derivative on equation 5a, we are able to use that equation to calculate the value for the character at time t + 1:

$$\bar{n}_{t+1} = \bar{n}_t + \Gamma_n [\partial \ln W_n / \partial \bar{n}] \quad (6a)$$

```
#Calculate the value for the host character for t + 1
n_t1 <- n_t + (AgH/(N_t1/N_t)) * eq1_D(n_t)

# d ln (P_t1/N_t) / d p_t -> 1/(P_t1/N_t) * d (P_t1/P_t) / d p_t
# the change on the fitness of the parasitoid P / the change on the parasitoid P
character

eq2_D <-function (p_deriv)
{
.e1 <- 1 + N_t * (Lambda - 1)/K
.e2 <- n_t - p_deriv
.e3 <- .e1 * (n_p - C_p * p_deriv)
.e4 <- exp(-(s * .e2^2))
.e6 <- 1 + a_p * P_t * (1 + a_p * N_t * .e4/.e3)/k_p
c_p * N_t * (2 * (s * (1 - 1/.e6^k_p) * .e2) + a_p^2 * N_t *
P_t * (2 * (s * .e2/.e3) + C_p * .e1/.e3^2) * .e4/.e6^(1 +
k_p)) * .e4/(P_t * .e1)
}

#Calculate the value for the host character for t + 1
p_t1 <- p_t + (AgP/(P_t1/P_t)) * eq2_D(p_t)
```

With the following script we save the results for using in the next time step of the loop.

```
#Save all results for next gtime step
Results_table$H_density[t+1] <- N_t1
Results_table$P_density[t+1] <- P_t1
Results_table$H_character[t+1] <- n_t1
Results_table$P_character[t+1] <- p_t1
Results_table$Parasitism_rate_P[t+1]<-(1-alphaM)*(1-f(N_t,P_t))
Results_table$Proportion_P[t+1]<-(1-f(N_t,P_t))
Results_table$alpha_value_P[t+1] <- alphaM
```

```
}

#here is the end of the simulations
return(Results_table)
}
```

Before running the model, we need to set the initial parameters for the model. Below is one example of asigning values to all the parameters in the model:

```
##Parameters for the ecological part of the model
#Host growth rate
Lambda <- 20

#Host carrying capacity
K <- 700

# Intrinsic attack rate of parasitoid for "P"
n_p <- 37

# Mean survival of parasitoid female larvae per parasitized host for "P"
c_p <- 0.92

# Clumping parameter of the negative binomial distribution for "P"
k_p <- 0.11

# Search efficiency for "P"
a_p <- 0.33

# Initial host density
N_0 <- K

# Initial parasitoid density for "P"
P_0 <- 10

##Parameters for the co-evolutionary part of the model
#Additive genetic variance in Host
AgH <- 0.01

#Additive genetic variance in Parasitoid for "P"
AgP <- c(0,0.01)

#Cost to host character
C_n <- 1

#Cost to parasitoid character for "P"
C_p <- 1

#Inflection to refuge function (scale factor)
s <- 1

#Initial host character
n_0 <- 1

#Initial parasitoid character for "P"
p_0 <- 0.9
```

And here we run the model as a function

```
#Run the model function with this parameters
CoEvoModel_Two_table(Lambda,K, n_p,c_p, k_p,a_p,N_0,P_0,AgH,
AgP,C_n,C_p,s,n_0,p_0)
```

Extension of the Two-species model to the Three-species model - R script with comments.

```
#Co-Evolutionary model adapted from Hochberg and Holt, 1995
#Three-species model, one host (N) and two parasitoids (P and W)

CoEvoModel_Three_table <-
function(Lambda,K,n_p,n_y,c_p,c_y,k_p,k_y,a_p,a_y,N_0,P_0,Y_0,AgH,AgP,AgY,C_n,C_
p,C_y,s,n_0,p_
0,y_0)
{

#Maximum gtime that the simulation will run for
gtime <- 50000

#Table to save the results of the model

Results_table <- data.frame(timestep= numeric(gtime), H_density= numeric(gtime),
P_density = numeric(gtime), Y_density= numeric(gtime), H_character=
numeric(gtime), P_character= numeric(gtime),Y_character= numeric(gtime),
Proportion_P= numeric(gtime), Proportion_Y= numeric(gtime), Parasitism_rate_P=
numeric(gtime), Parasitism_rate_Y = numeric(gtime), alpha_value_P=
numeric(gtime), alpha_value_Y= numeric(gtime))

Results_table[Results_table == 0] <- as.integer("")

Results_table$timestep <- c(1:gtime)

#Initial values for density and characters
#Only initial values for the first parasitoid and host are introduced at this
point

#Initial value for host density
Results_table$H_density[1] <- N_0

#Initial value for the first parasitoid density
Results_table$P_density[1] <- P_0

#Initial value for the host character
Results_table$H_character[1] <- n_0

#Inicial value for the first parasitoid character
#(host character >= parasitoid character is a constraint of the model)

Results_table$P_character[1] <- p_0

#Here starts the loop for the first 1/100 of the generation gtime
#for a two species model (we want to have a two species model run before we
introduce a second parasitoid)

for(t in 1:500) {

#Function for the proportion of host not available for the parasitoid (in a
refuge) in generation t
```

```
alpha_f <- function(n,x){1 - exp(-s*(n-x)^2)}

#Function for the host realized intrinsec growth rate in generation t
#This Lambda is not included in the density dependent survival of equetion
g(N_t)

Lambda_f <- function(n){Lambda - C_n*n}

#Function for the realized intrinsec attack rate for the parasitoid
#Here x is the character value in gtime t for a given parasitoid
#n_x is the original attack rate for the parasitoid (it can be n_p or n_y)

Attack_f <- function(n_x,x)(n_x - C_p*x)

#Read the values for the densities and character values from this gtime step
N_t <- Results_table$H_density[t]
P_t <- Results_table$P_density[t]
n_t <- Results_table$H_character[t]
p_t <- Results_table$P_character[t]

#Calculate the realized growth rate for the host inside of the refuge
LambdaM <- Lambda_f(n_t)

#Calculate the realized attack rate for the parasitoid
n_p_M <- Attack_f(n_p,p_t)

#Calculate the proportion of host in the refuge
alphaM <- alpha_f(n_t,p_t)

#Proportion of host escaping density dependent mortality
g<-function(N){(1 + N * (Lambda - 1)/K)^-1}

#Proportion of host OUTSIDE the refuge that survive parasitism
#Note that the cost to the attack rate of the parasitoid is included in the
equation already = n_p - C_p*p_t

f<-function(N,P){(1 + (a_p * P)/(k_p * (1 + a_p * N * g(N) * (1 - alphaM)/(n_p-
C_p*p_t))))^-k_p}

####################################################################
#Basic ecological model
#Note that the co-evolutionary part of the model is included in f(N_t,P_t), in
LambdaM and in alphaM

#Density of host at gtime t+1
N_t1 <- LambdaM * N_t * g(N_t) * (alphaM + (1-alphaM) * f(N_t,P_t))

#Density of parasitoid at t+1
P_t1 <- c_p * N_t * g(N_t) * (1-alphaM) * (1-f(N_t,P_t))

#Evolution of phenotypic means
# d ln (N_t1/N_t) / d n_t -> 1/(N_t1/N_t) * d (N_t1/N_t) / d n_t
# the change on the fitness of the host / the change on the host character

eq1_D <- function (n_deriv)
{
.e1 <- n_deriv - p_t
```

```
.e2 <- exp(-(s * .e1^2))
.e3 <- 1 + N_t * (Lambda - 1)/K
.e5 <- n_p - C_p * p_t
.e7 <- 1 + a_p * P_t * (1 + a_p * N_t * .e2/(.e3 * .e5))/k_p
.e9 <- 1/.e7^k_p - 1
(s * (2 * (a_p^2 * N_t * P_t * .e2/(.e7^(1 + k_p) * .e3 *
.e5)) - 2 * .e9) * .e2 * (Lambda - C_n * n_deriv) * .e1 -
C_n * (1 + .e9 * .e2))/.e3
}

#Calculate the value for the host character for t + 1

n_t1 <- n_t + (AgH/(N_t1/N_t)) * eq1_D(n_t)

# d ln (P_t1/N_t) / d p_t -> 1/(P_t1/N_t) * d (P_t1/P_t) / d p_t
# the change on the fitness of the parasitoid P / the change on the parasitoid P
character

eq2_D <-function (p_deriv)
{
.e1 <- 1 + N_t * (Lambda - 1)/K
.e2 <- n_t - p_deriv
.e3 <- .e1 * (n_p - C_p * p_deriv)
.e4 <- exp(-(s * .e2^2))
.e6 <- 1 + a_p * P_t * (1 + a_p * N_t * .e4/.e3)/k_p
c_p * N_t * (2 * (s * (1 - 1/.e6^k_p) * .e2) + a_p^2 * N_t *
P_t * (2 * (s * .e2/.e3) + C_p * .e1/.e3^2) * .e4/.e6^(1 +
k_p)) * .e4/(P_t * .e1)
}

#Calculate the value for the host character for t + 1
p_t1 <- p_t + (AgP/(P_t1/P_t)) * eq2_D(p_t)

#Save all results for next gtime step

Results_table$H_density[t+1] <- N_t1
Results_table$P_density[t+1] <- P_t1
Results_table$H_character[t+1] <- n_t1
Results_table$P_character[t+1] <- p_t1
Results_table$Parasitism_rate_P[t+1]<-(1-alphaM)*(1-f(N_t,P_t))
Results_table$Proportion_P[t+1]<-(1-f(N_t,P_t))
Results_table$alpha_value_P[t+1] <- alphaM
}

################################################################################
#########
#From this point forward, another Parasitoid is introduced to the system -
Parasitoid Y
#Initial value for the second parasitoid density - Y

Results_table$Y_density[500] <- Y_0

#Inicial value for the second parasitoid character
#(host character >= parasitoid character is a constraint of the model)

Results_table$Y_character[500] <- y_0
```

```
for(t in 500:(gtime-1))
{

##Here we make the desition based on the density of the parasitoids if the
parasitoids are extict or not
##Based on this we chose to continue with a "two species model" or a "three
species model".
##See Figure B1.
```

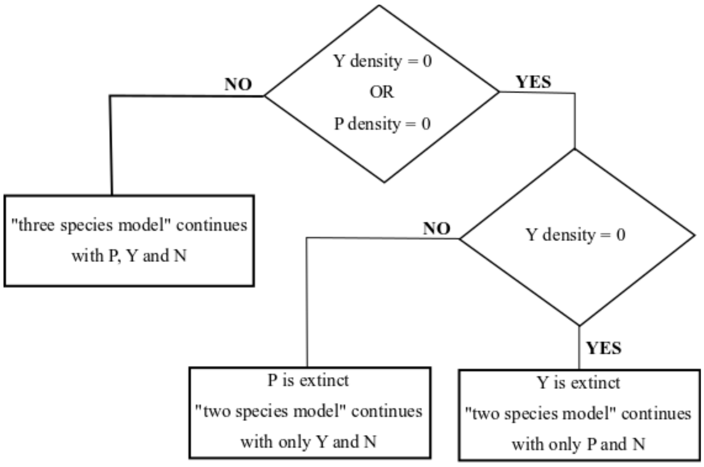

Figure A. Diagram of the decision making process for continue with the "three species model" or with a "two species model" during the simulation.

```
if(Results_table$Y_density[t]==0 | Results_table$P_density[t]==0 |
is.na(Results_table$Y_density[t]) |
is.na(Results_table$P_density[t]))
{
if(Results_table$Y_density[t]==0)
{

#Parasitoid Y went extinct, and for the rest of the simulation the model is a
two species model
#with host N and parasitoid P

N_t <- Results_table$H_density[t]
P_t <- Results_table$P_density[t]
n_t <- Results_table$H_character[t]
p_t <- Results_table$P_character[t]

#Calculate cost lambda for the host
LambdaM <- Lambda_f(n_t)

#Calculate the proportion of host in the refuge
alphaM <- alpha_f(n_t,p_t)

#Density dependent survival
g<-function(N){(1 + N * (Lambda - 1)/K)^-1}

#Proportion of host outside the refuge that survive parasitism
f<-function(N,P){(1 + (a_p * P)/(k_p * (1 + a_p * N * g(N) * (1 - alphaM)/(n_p-
C_p*p_t))))^-k_p}

######################################################################
#Two species model
```

```
#Host density in t + 1

N_t1 <- LambdaM * N_t * g(N_t) * (alphaM + ((1-alphaM) * f(N_t,P_t)))

#Parasitoid P density in t + 1
P_t1 <- c_p * N_t * g(N_t) * (1-alphaM) * (1-f(N_t,P_t))

#Changes in fitness with the changes in character of the host
eq1_D <- function (n_deriv)
{
.e1 <- n_deriv - p_t
.e2 <- exp(-(s * .e1^2))
.e3 <- 1 + N_t * (LambdaM - 1)/K
.e5 <- n_p - C_p * p_t
.e7 <- 1 + a_p * P_t * (1 + a_p * N_t * .e2/(.e3 * .e5))/k_p
.e9 <- 1/.e7^k_p - 1
(s * (2 * (a_p^2 * N_t * P_t * .e2/(.e7^(1 + k_p) * .e3 *
.e5)) - 2 * .e9) * .e2 * (Lambda - C_n * n_deriv) * .e1 -
C_n * (1 + .e9 * .e2))/.e3
}

#Calculate the character value for the host at t + 1

n_t1 <- n_t + (AgH/(N_t1/N_t)) * eq1_D(n_t)

#Changes in fitness with the changes in character of the parasitoid

eq2_D <-function (p_deriv)
{
.e1 <- 1 + N_t * (Lambda - 1)/K
.e2 <- n_t - p_deriv
.e3 <- .e1 * (n_p - C_p * p_deriv)
.e4 <- exp(-(s * .e2^2))
.e6 <- 1 + a_p * P_t * (1 + a_p * N_t * .e4/.e3)/k_p
c_p * N_t * (2 * (s * (1 - 1/.e6^k_p) * .e2) + a_p^2 * N_t *
P_t * (2 * (s * .e2/.e3) + C_p * .e1/.e3^2) * .e4/.e6^(1 +
k_p)) * .e4/(P_t * .e1)
}

#Calculate the character value for the host at t + 1

p_t1 <- p_t + (AgP/(P_t1/P_t)) * eq2_D(p_t)

#Save results for next time step

Results_table$H_density[t+1] <- N_t1
Results_table$P_density[t+1] <- P_t1
Results_table$Y_density[t+1] <- Results_table$Y_density[t]
Results_table$H_character[t+1] <- n_t1
Results_table$P_character[t+1] <- p_t1
Results_table$Parasitism_rate_P[t+1]<-(1-alphaM)*(1-f(N_t,P_t))
Results_table$Proportion_P[t+1]<-(1-f(N_t,P_t))
Results_table$alpha_value_P[t+1] <- alphaM
}else{

#Parasitoid P goes extinct and the model continuous as a two species model with
the host N and Parasitoid Y
```

```
N_t <- Results_table[t,2]
Y_t <- Results_table[t,4]
n_t <- Results_table[t,5]
y_t <- Results_table[t,7]

#Calculate Lambda for this timestep

LambdaM <- Lambda_f(n_t)

#Calculate alpha for this timestep

alphaM <- alpha_f(n_t,y_t)

#Density dependent survival

g<-function(N){(1 + N * (Lambda - 1)/K)^-1}

#Proportion of host surviving parasitism

h<-function(N,Y){(1 + (a_y * Y)/(k_y * (1 + a_y * N * g(N) * (1 - alphaM)/(n_y-
C_y*y_t))))^-k_y}

####################################################################
#Two species model
#Host density in t + 1

N_t1 <- LambdaM * N_t * g(N_t) * (alphaM + ((1-alphaM) * h(N_t,Y_t)))

#Parasitoid Y densiti in t + 1

Y_t1 <- c_y * N_t * g(N_t) * (1-alphaM) * (1-h(N_t,Y_t))

#Changes in fitness with the changes in character of the host

eq1_D <-function (n_deriv)
{
.e1 <- n_deriv - y_t
.e2 <- exp(-(s * .e1^2))
.e3 <- 1 + N_t * (Lambda - 1)/K
.e5 <- n_y - C_y * w_t
.e7 <- 1 + a_y * Y_t * (1 + a_y * N_t * .e2/(.e3 * .e5))/k_y
.e9 <- 1/.e7^k_y - 1
(s * (2 * (a_y^2 * N_t * Y_t * .e2/(.e7^(1 + k_y) * .e3 *
.e5)) - 2 * .e9) * .e2 * (Lambda - C_n * n_deriv) * .e1 -
C_n * (1 + .e9 * .e2))/.e3
}

#Calculate the character value for the host at t + 1

n_t1 <- n_t + (AgH/(N_t1/N_t)) * eq1_D(n_t)

#Changes in fitness with the changes in character of the parasitoid Y

eq2_D <-function (w_deriv)
{
.e1 <- 1 + N_t * (Lambda - 1)/K
.e2 <- y_t - y_deriv
.e3 <- .e1 * (n_y - C_y * y_deriv)
```

```
.e4 <- exp(-(s * .e2^2))
.e6 <- 1 + a_y * Y_t * (1 + a_y * N_t * .e4/.e3)/k_y
c_y * N_t * (2 * (s * (1 - 1/.e6^k_y) * .e2) + a_y^2 * k_y *
N_t * Y_t * (2 * (s * .e2/.e3) + C_y * .e1/.e3^2) * .e4/(k_p *
.e6^(1 + k_y))) * .e4/(Y_t * .e1)
}

#Calculate the character value for the Parasitoid Y at t + 1

y_t1 <- y_t + (AgY/(Y_t1/Y_t)) * eq2_D(y_t)

#Save results for next time step

Results_table$H_density[t+1] <- N_t1
Results_table$Y_density[t+1] <- Y_t1
Results_table$P_density[t+1] <- Results_table$P_density[t]
Results_table$H_character[t+1] <- n_t1
Results_table$Y_character[t+1] <- y_t1
Results_table$Parasitism_rate_Y[t+1] <- (1-alphaM)*(1-h(N_t,Y_t))
Results_table$Proportion_Y[t+1]<- (1-h(N_t,Y_t))
Results_table$alpha_value_Y[t+1] <- alphaM
}
}else{

###############################################################################
###
#Three species model

N_t <- Results_table$H_density[t]
P_t <- Results_table$P_density[t]
Y_t <- Results_table$Y_density[t]
n_t <- Results_table$H_character[t]
p_t <- Results_table$P_character[t]
y_t <- Results_table$Y_character[t]

#Calculate growth rate for the host inside the refuge

LambdaM <- Lambda_f(n_t)

#Calculate the proportion of host not available for each parasitoid

alpha_P <- alpha_f(n_t,p_t)
alpha_Y <- alpha_f(n_t,y_t)

#Host density dependent survival

g<-function(N){(1 + N * (Lambda - 1)/K)^-1}

#Proportion of host surviving from Parasitoid P

f<-function(N,P){(1 + (a_p * P)/(k_p * (1 + a_p * N * g(N) * (1 – alpha_P)/(n_p-
C_p*p_t))))^-k_p}

#Proportion of host surviving from Parasitoid Y

h<-function(N,Y){(1 + (a_y * Y)/(k_y * (1 + a_y * N * g(N) * (1 - alpha_Y)/(n_y-
C_y*y_t))))^-k_y}
```

```
#Density of host at t + 1

N_t1 <- LambdaM * N_t * g(N_t) * ((alpha_P + (1-alpha_P) * f(N_t,P_t)) *
(alpha_Y + (1-alpha_Y) * h(N_t,Y_t)))

#Density of Parasitoid Y at t + 1
Y_t1 <- c_y * N_t * g(N_t) * (1-alpha_Y) * (1-h(N_t,Y_t)) * f(N_t,P_t)

#Density of Parasitoid P at t + 1
P_t1 <- c_p * N_t * g(N_t) * (1-alpha_P) * (1-f(N_t,P_t)) * h(N_t,Y_t)

eq1_D <- function (n_deriv)
{
.e1 <- LambdaM - 1
.e2 <- n_deriv - p_t
.e3 <- n_deriv - y_t
.e5 <- exp(-(s * .e2^2))
.e6 <- exp(-(s * .e3^2))
.e7 <- 1 + N_t * .e1/K
.e8 <- 1 + Y_t * .e1/K
.e11 <- n_p - C_p * p_t
.e12 <- n_y - C_y * y_t
.e14 <- 1 + a_p * P_t * (1 + a_p * N_t * .e5/(.e7 * .e11))/k_p
.e15 <- 1 + a_y * Y_t * (1 + a_y * Y_t * .e6/(.e8 * .e12))/k_y
.e17 <- 1/.e14^k_p - 1
.e19 <- 1/.e15^k_y - 1
.e20 <- 1 + .e17 * .e5
.e21 <- 1 + .e19 * .e6
(s * (.e20 * (2 * (a_y^2 * Y_t^2 * .e6/(.e15^(1 + k_y) *
.e8 * .e12)) - 2 * .e19) * .e6 * .e3 + .e21 * (2 * (a_p^2 *
N_t * P_t * .e5/(.e14^(1 + k_p) * .e7 * .e11)) - 2 *
.e17) * .e5 * .e2) * (Lambda - C_n * n_deriv) - C_n *
.e20 * .e21)/.e7
}

n_t1 <- n_t + (AgH/(N_t1/N_t)) * eq1_D(n_t)

eq2_D <- function (p_deriv)
{
.e1 <- 1 + N_t * (Lambda - 1)/K
.e2 <- n_t - p_deriv
.e3 <- .e1 * (n_p - C_p * p_deriv)
.e4 <- exp(-(s * .e2^2))
.e6 <- 1 + a_p * P_t * (1 + a_p * N_t * .e4/.e3)/k_p
c_p * N_t * (2 * (s * (1 - 1/.e6^k_p) * .e2) + a_p^2 * N_t *
P_t * (2 * (s * .e2/.e3) + C_p * .e1/.e3^2) * .e4/.e6^(1 +
k_p)) * .e4/(P_t * (1 + a_y * Y_t * (1 + a_y * N_t *
exp(-(s * (n_t - y_t)^2))/(.e1 * (n_y - C_y * y_t)))/k_y)^k_y *
.e1)
}

p_t1 <- p_t + (AgP/(P_t1/P_t)) * eq2_D(p_t)

eq3_D <- function (y_deriv)
{
.e1 <- 1 + N_t * (Lambda - 1)/K
.e2 <- n_t - y_deriv
.e3 <- .e1 * (n_y - C_y * y_deriv)
.e4 <- exp(-(s * .e2^2))
.e6 <- 1 + a_y * Y_t * (1 + a_y * N_t * .e4/.e3)/k_y
```

```
c_y * N_t * (2 * (s * (1 - 1/.e6^k_y) * .e2) + a_y^2 * N_t *
Y_t * (2 * (s * .e2/.e3) + C_y * .e1/.e3^2) * .e4/.e6^(1 +
k_y)) * .e4/(Y_t * (1 + a_p * P_t * (1 + a_p * N_t *
exp(-(s * (n_t - p_t)^2))/(.e1 * (n_p - C_p * p_t)))/k_p)^k_p *
.e1)
}

y_t1 <- y_t + (AgY/(Y_t1/Y_t)) * eq3_D(y_t)

#Save the results for the next time step

Results_table$H_density[t+1] <- N_t1
Results_table$P_density[t+1] <- P_t1
Results_table$Y_density[t+1] <- Y_t1
Results_table$H_character[t+1] <- n_t1
Results_table$P_character[t+1] <- p_t1
Results_table$Y_character[t+1] <- y_t1
Results_table$Parasitism_rate_P[t+1]<-(1-alpha_P)*(1-f(N_t,P_t))
Results_table$Parasitism_rate_Y[t+1]<-(1-alpha_Y)*(1-h(N_t,Y_t))
Results_table$Proportion_P[t+1]<-(1-f(N_t,P_t))
Results_table$Proportion_Y[t+1]<-(1-h(N_t,Y_t))
Results_table$alpha_value_P[t+1] <- alpha_P
Results_table$alpha_value_Y[t+1] <- alpha_Y
}
}
return(Results_table)
}

#PARAMETERS

#Host growth rate
Lambda <- 20

#Host carrying capacity
K <- 700

# Intrinsic attack rate of parasitoid for "P"
n_p <- 50

# Intrinsic attack rate of parasitoid for "Y"
n_y <- 25

# Mean survival of parasitoid female larvae per parasitized host for "P"
c_p <- 0.92

# Mean survival of parasitoid female larvae per parasitized host for "Y"
c_y <- 0.92

# Clumping parameter of the negative binomial distribution for "P"
k_p <- 0.5

# Clumping parameter of the negative binomial distribution for "Y"
k_y <- 0.5

# Search efficiency for "P"
a_p <- 0.25

# Search efficiency for "Y"
a_y <- 0.25
```

```
# Initial host density
N_0 <- K
# Initial parasitoid density for "P"
P_0 <- 10

# Initial parasitoid density for "Y"
Y_0 <- 10

##Parameters for the co-evolutionary model
#Additive genetic variance in Host
AgH <- 0.01

#Additive genetic variance in Parasitoid for "P"
AgP <- 0

#Additive genetic variance in Parasitoid for "Y"
AgY <- 0.01

#Cost to host character
C_n <- 1

#Cost to parasitoid character for "P"
C_p <- 1

#Cost to parasitoid character for "Y"
C_y <- 1

#Initial host character
n_0 <- 1

#Initial parasitoid character for "P"
p_0 <- 0.9

#Initial parasitoid character for "Y"
y_0 <- 0.9

#Run the model with above parameters

ResultsT <- CoEvoModel_Three_table(Lambda,K, n_p, n_y,c_p,c_y, k_p, k_y,a_p,a_y,N_0,P_0,Y_0,AgH,
AgP, AgY,C_n,C_p,C_y ,n_0, p_0, y_0)
```

Information supporting the values for the model parameters

Intrinsic growth rate of the host (λ) – We use data on λ from for *L. bonariensis* from the Waikato area [2]. We calculated λ for *S. discoideus* from life table data presented by [3] using the same method as Barker et al. (1989 [2]).

Host carrying capacity (adults/m2, K) – This is the maximum density found in the field before the introduction of the parasitoid. For L. bonariensis Barker et al. (1989 [2]) showed 720 m-2 at Waikato, and for S. discoideus, Goldson et al. (1984 [4]) found a maximum density of 773 m-2 at Canterbury.

Intrinsic attack rate of the parasitoid (η) – This was estimated as the maximum number of hosts each parasitoid female can attack if hosts are sufficiently numerous. Phillips et al. (1996 [5]) found that *M. hyperodae* can infect 42 hosts ± 22 (95% confidence interval) around Hammilton. For *M. aethiopoides,* Barlow and Goldson (1993 [6]) found the maximum rate of parasitoid increase per generation in Darfield to be 33. When working with the "three species model", we always set η of a "sexual" parasitoid to be half the value than η for an "asexual" parasitoid.

Survival of parasitoid larvae (c) – The mean survival of parasitoid female larvae perparasitized host, c, was calculated by Barratt et al. (2004 [7]) for *M. hyperodae* (0.92 with c. 8% eliciting host immune response). The equivalent data are unknown for *M. aethiopoides*, so we assumed the same value as for *M. hyperodae*.

Spatial heterogeneity in parasitism (κ) – This represents the clumping parameter of the negative binomial distribution, a measure of parasitism aggregation. As the value of κ increases the heterogeneity in parasitism decreases, small numbers of κ indicate aggregation in parasitism while bigger numbers approach a random distribution. For *L. bonariensis* and *M. hyperodae* we estimated the value of κ from data on parasitism rates at different parasitoid densities and different host densities from Barlow et al. (1993 [8]). The probability of escaping parasitism is described by the zero term in a negative bionomial distribution, with clumping parameter κ [9], therefore we calculated κ by fitting the data provided by Barlow et al. (1993 [8]) to the following equation: $Q = 1 - (1 + a \cdot P) - \kappa$. We used this equation and data on parasitoid density and parasitoid attacks from Barlow and Goldson (1993 [6]) to calculate κ for *M. aethiopoides* in lucerne.

References:

1. R Core Team. R: A Language and Environment for Statistical Computing [Internet]. Vienna, Austria: R Foundation for Statistical Computing; 2016. Available: https://www.R-project.org/

2. Barker GM, Pottinger RP, Addison PJ. Population dynamics of the argentine stem weevil (Listronotus bonariensis) in pastures of Waikato, New Zealand. Agric Ecosyst Environ. 1989;26: 79–115.

3. Goldson SL, Frampton ER, Proffitt JR. Population dynamics and larval establishment of Sitona discoideus (Coleoptera: Curculionidae) in New Zealand lucerne. J Appl Ecol. 1988; 177–195.

4. Goldson SL, Frampton ER, Barratt BIP, Ferguson CM. The seasonal biology of Sitona discoideus Gyllenhal (Coleoptera: Curculionidae), an introduced pest of New Zealand lucerne. Bull Entomol Res. 1984;74: 249–259.

5. Phillips CB, Baird DB. A morphometric method to assist in defining the South American origins of Microctonus hyperodae Loan (Hymenoptera: Braconidae) established in New Zealand. Biocontrol Sci Technol. 1996;6: 189–206.

6. Barlow ND, Goldson SL. A modelling analysis of the successful biological control of Sitona discoideus (Coleoptera: Curculionidae) by Microctonus aethiopoides (Hymenoptera: Braconidae) in New Zealand. J Appl Ecol. 1993;30: 165–178.

7. Barratt BIP. Chapter 9: Microctonus parasitoids and New Zealand Weevils: Comparing laboratory estimamates of host ranges to realized host ranges. In: Van Driesche RG, Murray T, Reardon R, editors. Assessing host ranges for parasitoids and predators used for classical biological control: a guide to best practice. Morgantown, West Virginia, USA: Forest Health Technology Enterprise Team; 2004.

8. Barlow ND, Goldson SL, McNeill MR, Proffitt JR. Measurement of the attack behaviour of Microctonus hyperodae as a classical biological control agent of Argentine stem weevil Listronotus bonaeriensis. 6th Australasian Grassland Invertebrate Ecological Conference. Hamilton, New Zealand; 1993. pp. 326–330.

9. May RM. Host-parasitoid systems in patchy environments: a phenomenological model. J Anim Ecol. 1978; 833–844.
