# Supplementary material for: Asymmetry in reproduction strategies drives evolution of resistance in biological control systems
Source: PLoS One. 2018 Dec 19;13(12):e0207610. doi: 10.1371/journal.pone.0207610 (PMC6300327; doi:10.1371/journal.pone.0207610)
Supplement: S2 File — (PDF) [file pone.0207610.s002.pdf]

S2 File. Results from the sensitivity analyses and figures illustrating the results from the "two-species model" and the "three species model".

Sensitivity analysis

Table A. Results for the sensitivity analysis of the two species model for 6000 simulations.

Parameter	Definition	Partial rank correlation coefficient	p-value
λ	Intrinsic growth rate of the host	-0.20	< 0.0001
K	Host carrying capacity (adults/m^2)	0.16	< 0.0001
η	Intrinsic rate of attack of the parasitoid	0.05	< 0.0001
c	Survival of parasitoid larvae	0.09	< 0.0001
κ	Spatial heterogeneity in parasitism	0.49	< 0.0001
Γ_n	Additive genetic variance of host	0.73	< 0.0001
Γ_p	Additive genetic variance of parasitoid	-0.71	< 0.0001
C_n	Cost to host character	0.10	< 0.0001
C_p	Cost to parasitoid character	0.001	0.909
N_0	Initial host density	0.02	0.063
P_0	Initial parasitoid density	0.01	0.248
n_0	Initial host character	-0.09	< 0.0001
p_0	Initial parasitoid character	0.11	< 0.0001

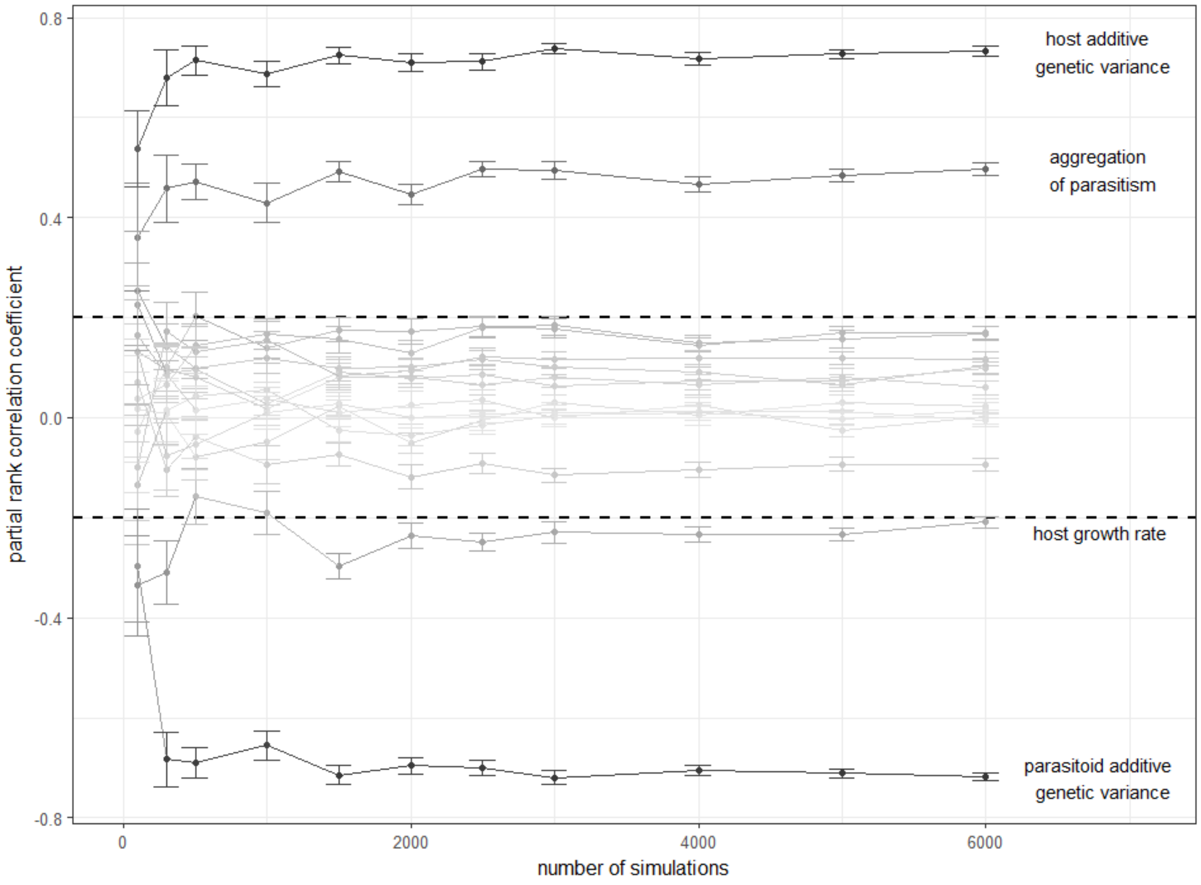

Fig A. Partial rank correlation coefficients (PRCC) from the sensitivity analysis of the two species co-evolutionary model. The dashed lines show the threshold for which we considered the parameters to have an important influence on the output of the model; an absolute value for the PRCC of 0.2 or higher. Only the four most important parameters are identified. Note how the values for the PRCC stabilize as the number of simulations increases.

Variation in results of the two species model with respect to the variation on the growth rate of the host (λ) and the spatial heterogeneity of parasitism (κ).

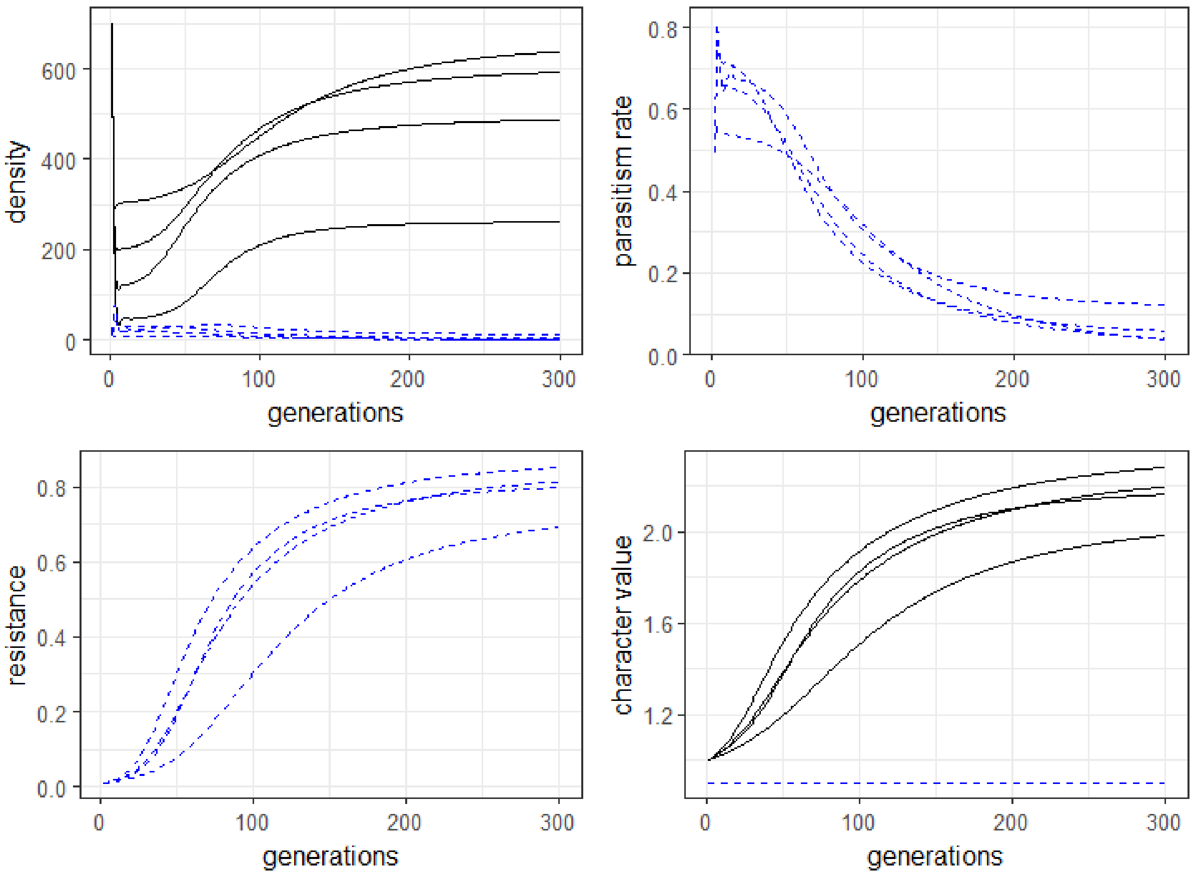

Fig B. Variation in the results when changing the growth rate of the host (λ), for a model with one host (black lines) and one **asexual** parasitoid (with no additive genetic variance, blue dashed lines). Four values of λ were used for this example (5, 10, 20 and 40). All the other parameters are the same in each simulation.

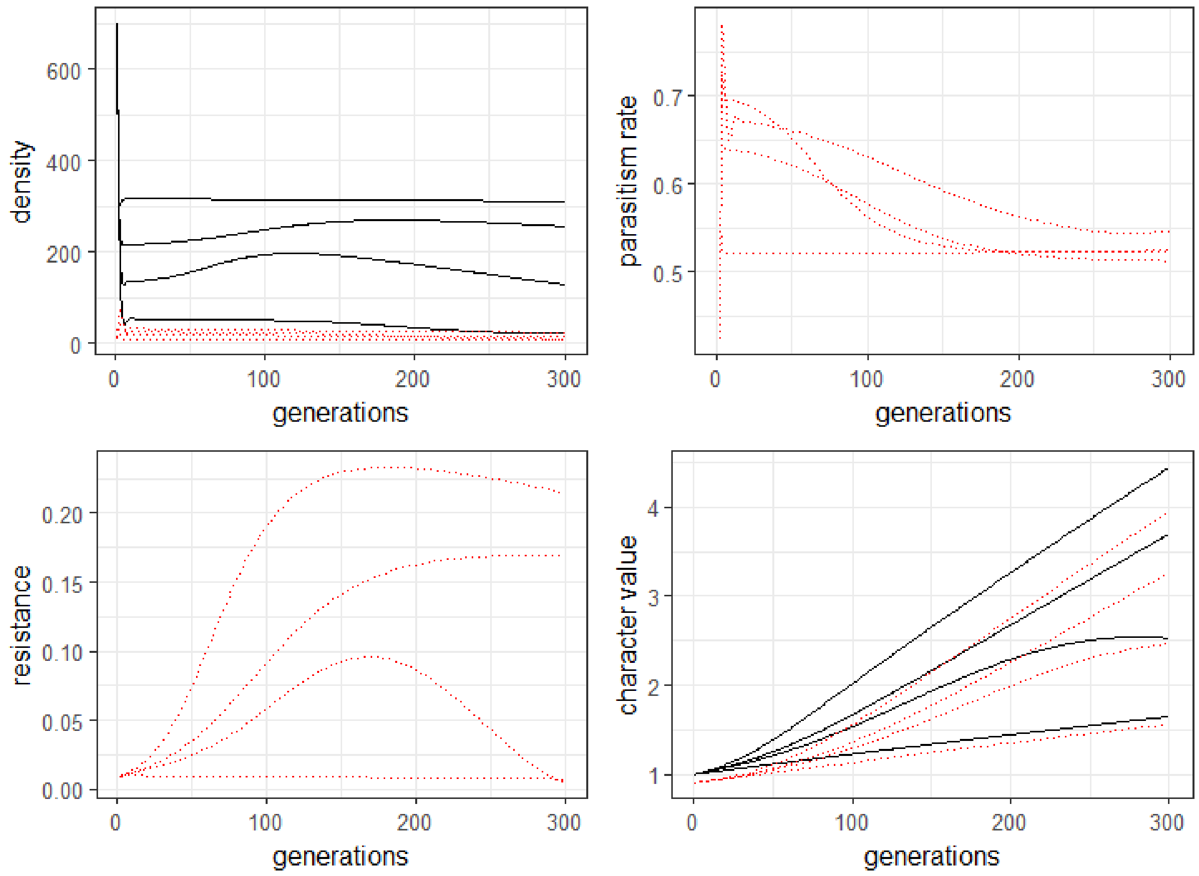

Fig C. Variation in the results when changing the growth rate of the host (λ), for a model with one host (black lines) and one **sexual** parasitoid (with additive genetic variance equal to the host, red dotted lines). Four values of λ were used for this example (5, 10, 20 and 40). All the other parameters are the same in each simulation.

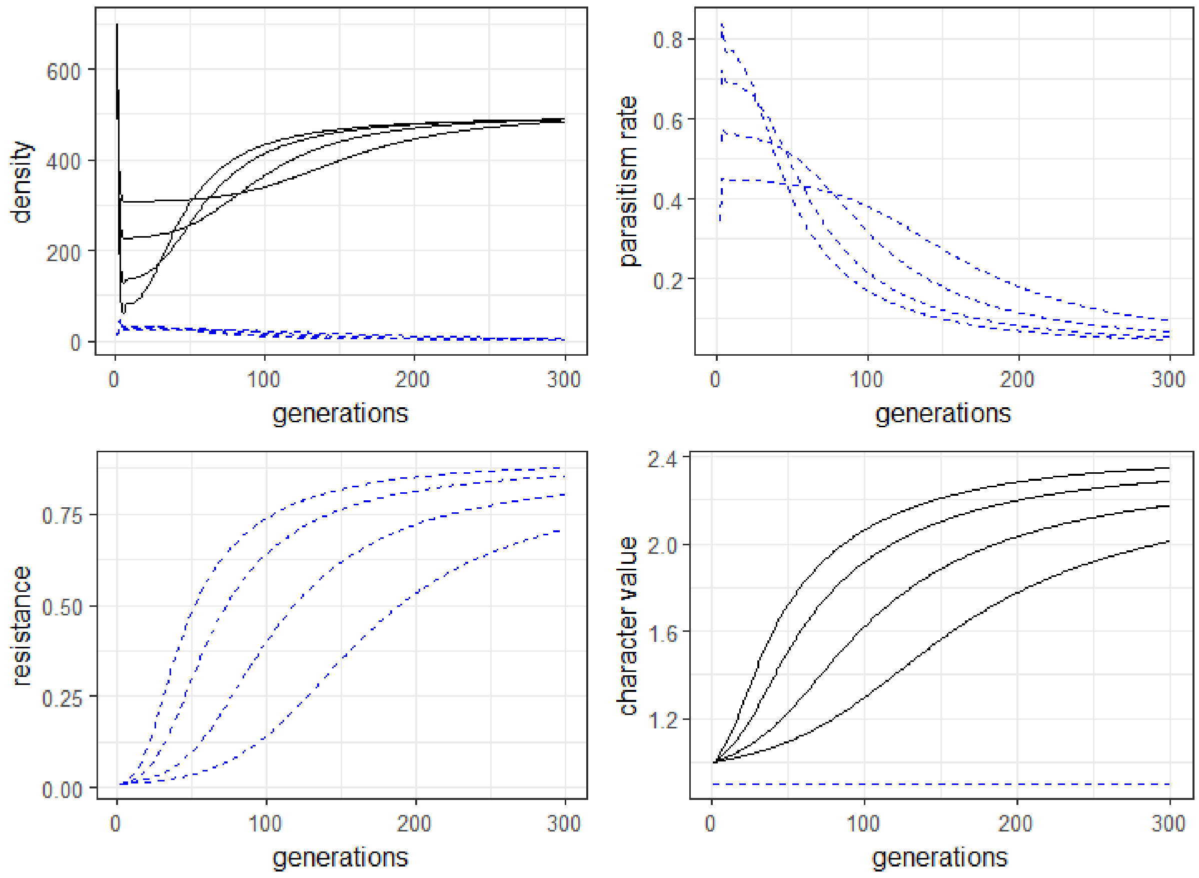

Fig D. Variation in the results when changing the spatial heterogeneity of parasitism (κ), for a model with one host (black lines) and one **asexual** parasitoid (with no additive genetic variance, blue dashed lines). Four values of κ were used for this example (0.1, 0.3, 0.5 and 0.8). All the other parameters are the same in each simulation.

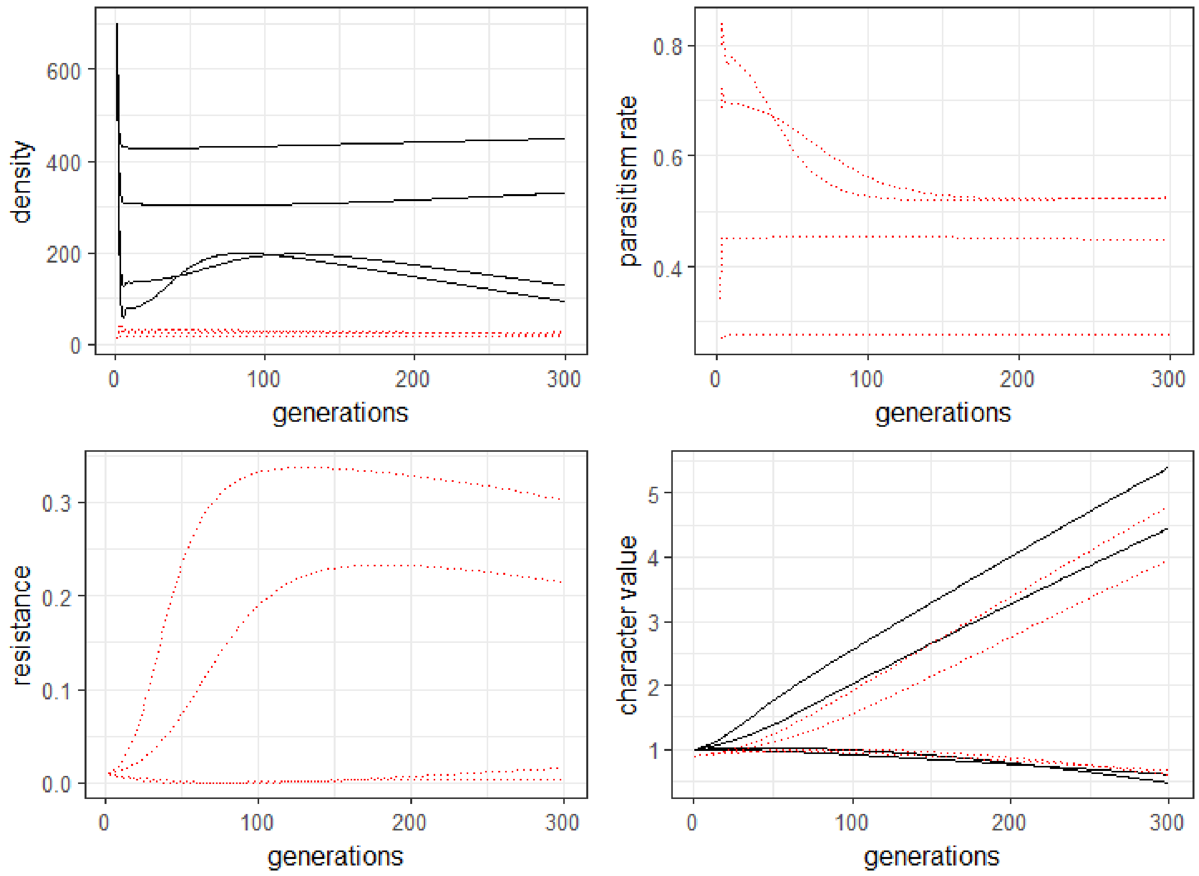

Fig S5. Variation in the results when changing the spatial heterogeneity in parasitism (κ), for a model with one host (black lines) and one **sexual** parasitoid (with additive genetic variance equal to the host, red dotted lines). Four values of κ were used for this example (0.1, 0.3, 0.5 and 0.8). All the other parameters are the same in each simulation.

Variation in results of the three species model with respect to the variation on the growth rate of the host (λ) and the spatial heterogeneity of parasitism (κ).

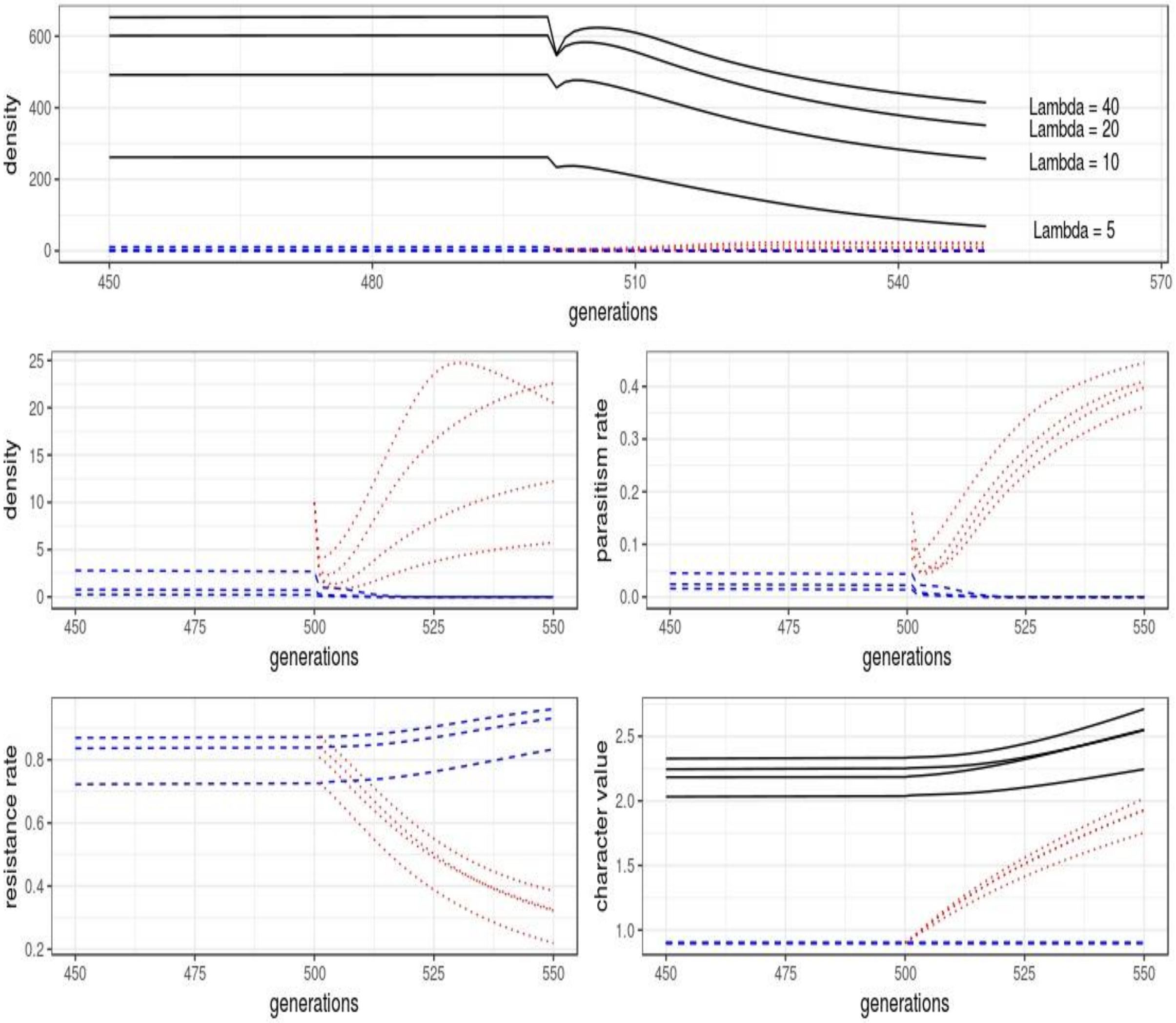

Fig E. An **asexual** parasitoid is introduced first with no additive genetic variance (blue dashed lines), followed by the introduction of a **sexual** parasitoid with additive genetic variance equal to the host, 0.01 (red dotted lines). Four values of λ were used for this example (5, 10, 20 and 40). The attack rate for the asexual parasitoid is double the attack rate for the sexual parasitoid (25 and 50 respectevily). Both parasitoids have the same value for the spatial heterogeneity in parasitism (0.5). The host is represented with black lines.

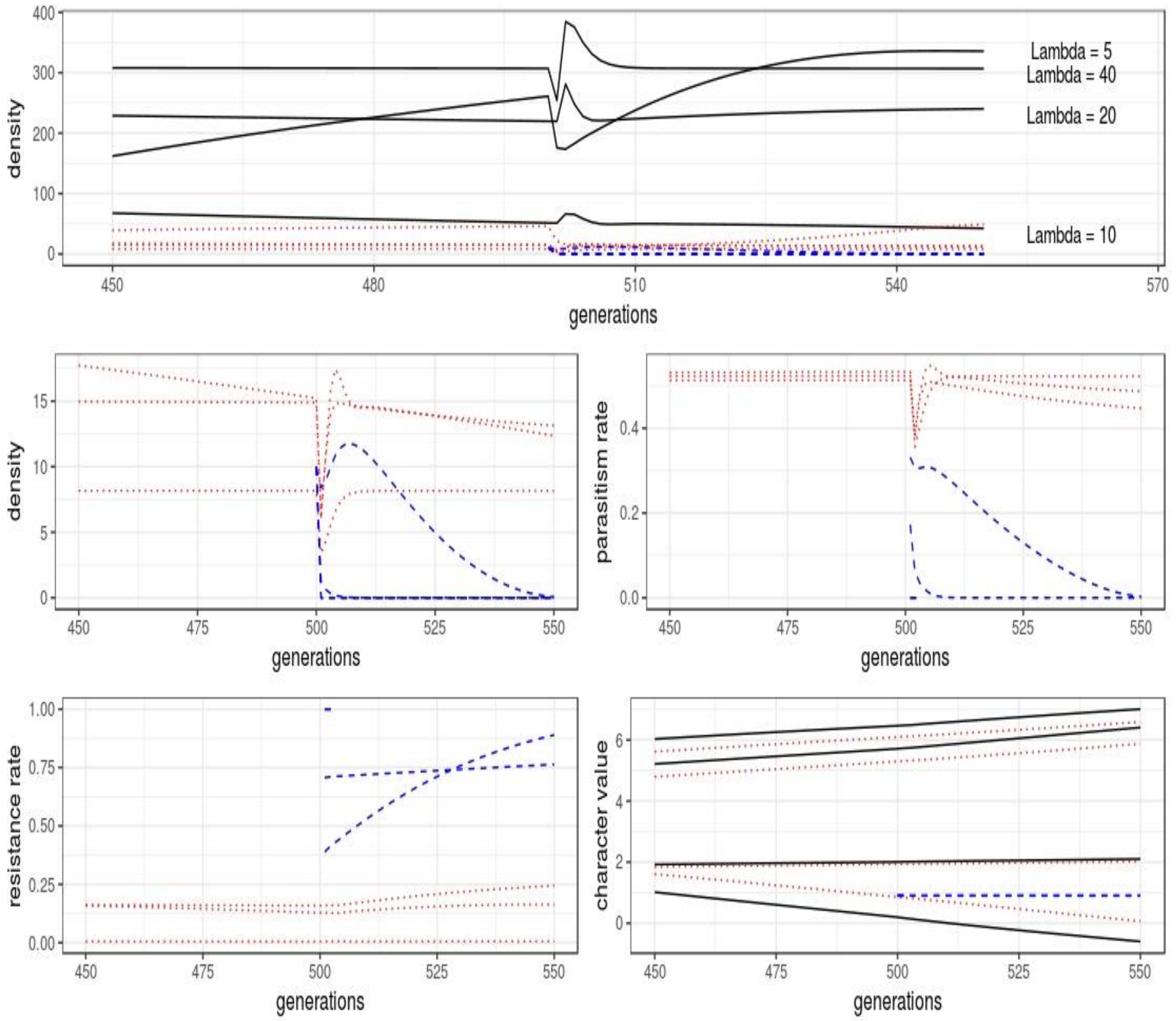

Fig F. A **sexual** parasitoid is introduced first with additive genetic variance equal to the host (red dotted lines), followed by the introduction of an **asexual** parasitoid with no additive genetic variance (blue dashed lines). Four values of λ were used for this example (5, 10, 20 and 40). The attack rate for the asexual parasitoid is double the attack rate for the sexual parasitoid (25 and 50 respectevily). Both parasitoids have the same value for the spatial heterogeneity in parasitism (0.5). The host is represented with black lines.

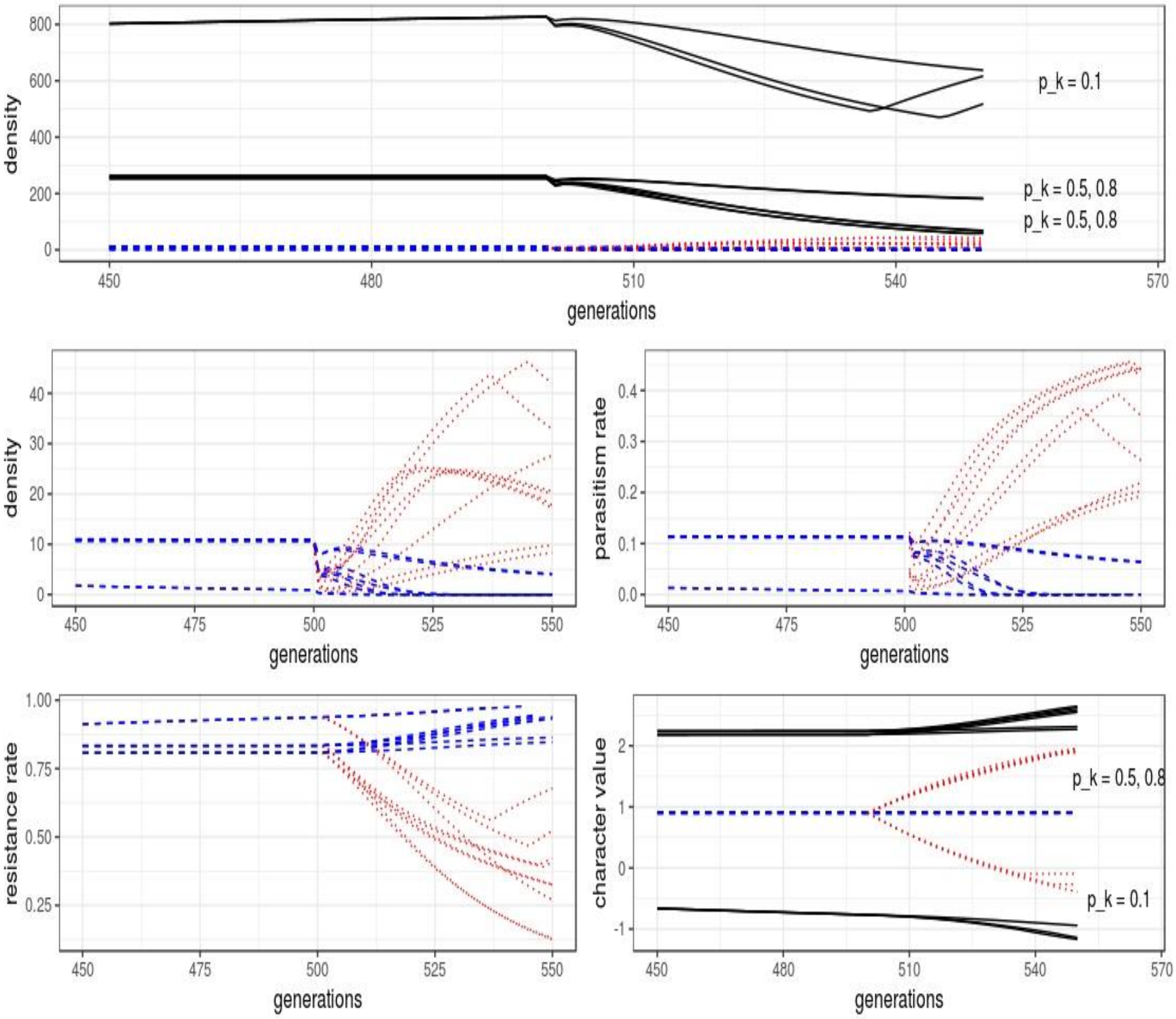

Fig G. An **asexual** parasitoid is introduced first with no additive genetic variance (blue dashed lines), followed by the introduction of a **sexual** parasitoid with additive genetic variance equal to the host, 0.01 (red dotted lines). Three values for the spatial heterogeneity in parasitism (κ) were used for this example (0.1, 0.5 and 0.8). The attack rate for the asexual parasitoid is double the attack rate for the sexual parasitoid (25 and 50 respectevily). The host is represented with black lines and its growth rate was 5 for all examples.

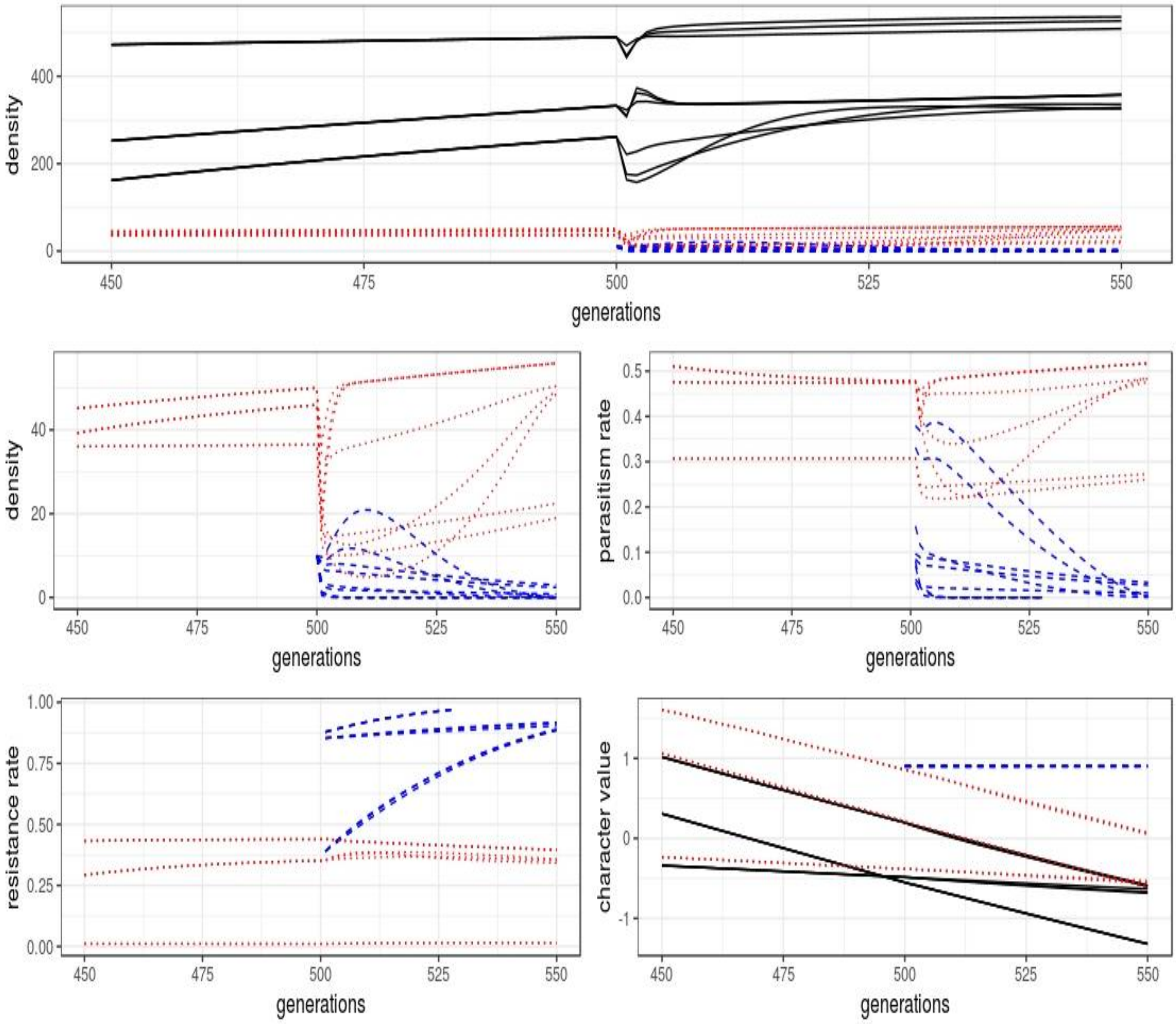

Fig H. A **sexual** parasitoid is introduced first with additive genetic variance equal to the host (red dotted lines), followed by the introduction of an **asexual** parasitoid with no additive genetic variance (blue dashed lines). Three values for the spatial heterogeneity in parasitism (κ) were used for this example (0.1, 0.5 and 0.8). The attack rate for the asexual parasitoid is double the attack rate for the sexual parasitoid (25 and 50 respectevily). The host is represented with black lines and its growth rate was 5 for all examples.

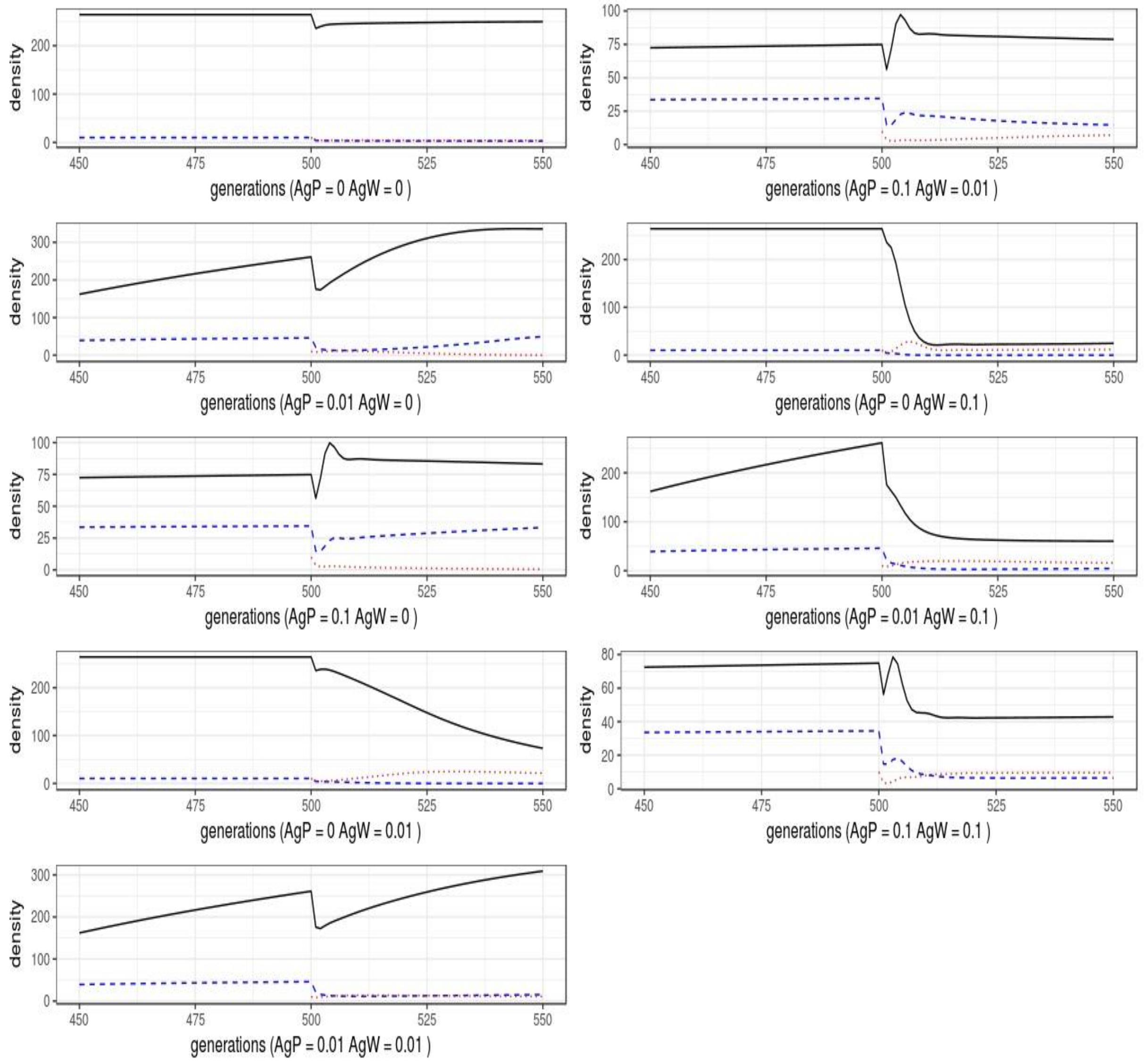

Fig I. Densities over time resulting from different combinations additive genetic variance of parasitoids for the three species model. The blue dashed line represents the parasitoid that is introduced first, and the red dotted line represents the parasitoid that is introduced second. “AgP” is the additive genetic variance of the first parastitoid, and “AgW” is the additive genetic variance of the second parasitoid.

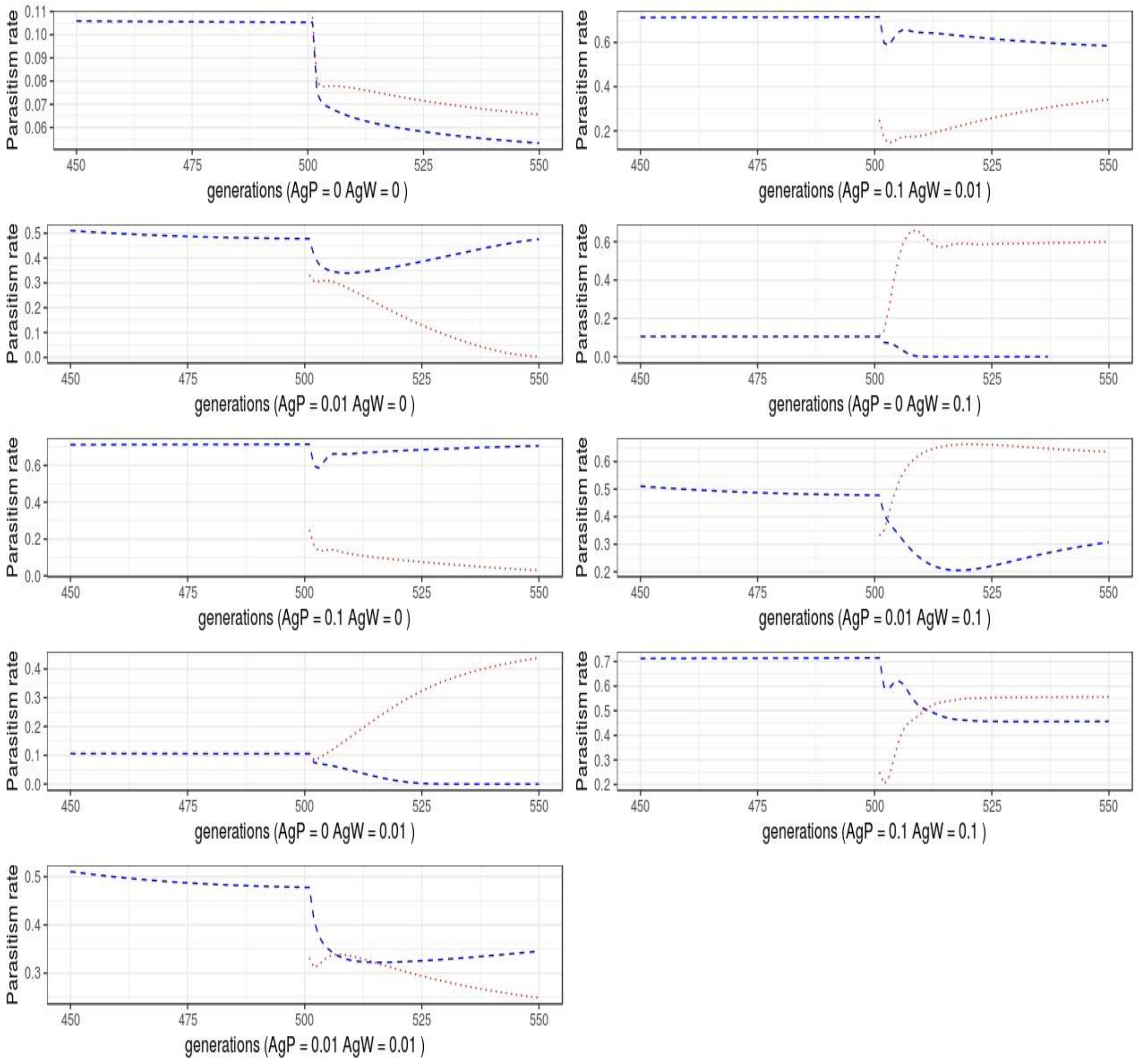

Fig J. Parasitism rate over time resulting from different combinations additive genetic variance of parasitoids for the three species model. The blue dashed line represents the parasitoid that is introduced first, and the red dotted line represents the parasitoid that is introduced second. “AgP” is the additive genetic variance of the first parastitoid, and “AgW” is the additive genetic variance of the second parasitoid.

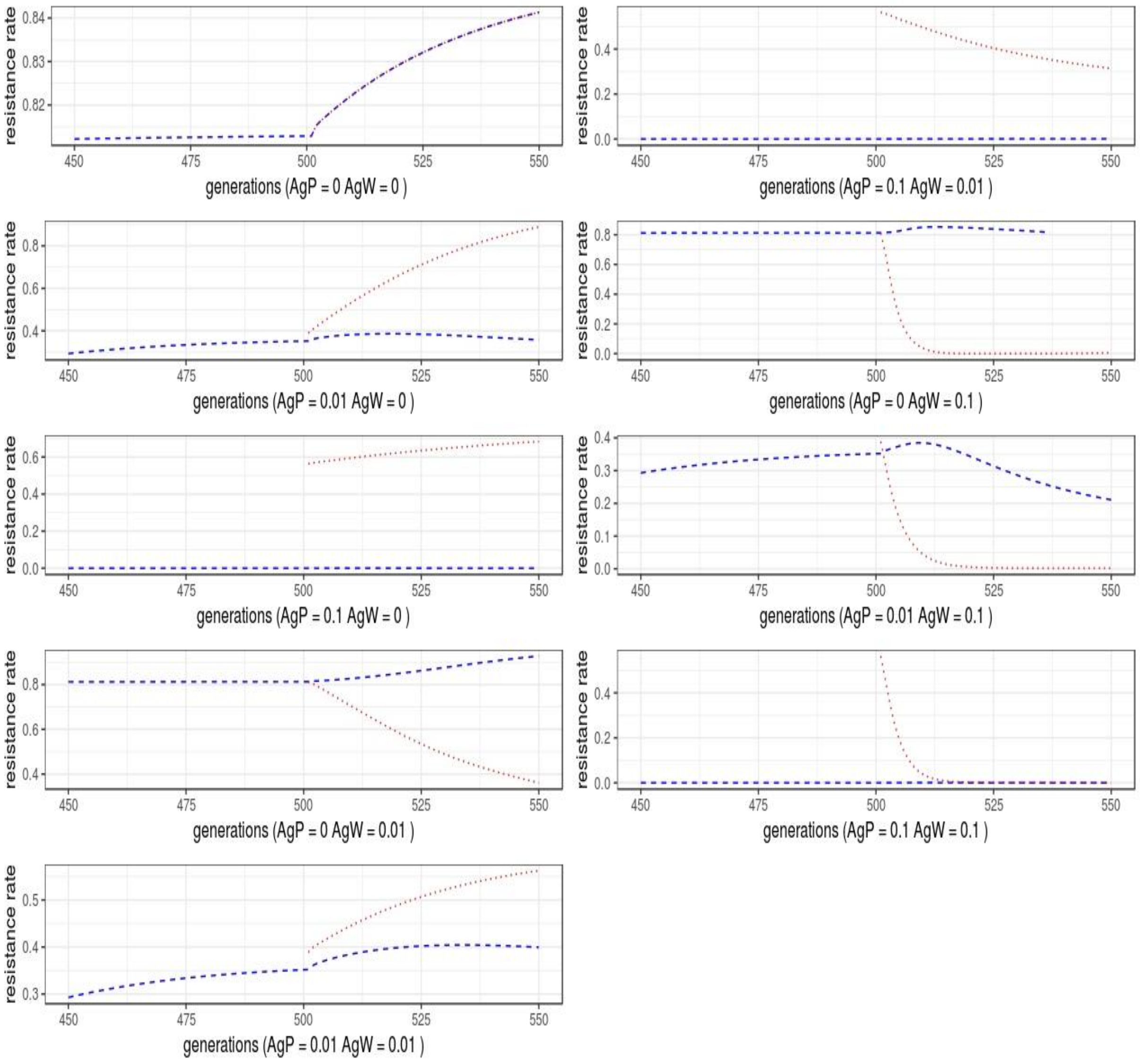

Fig K. Proportion of resistant host over time resulting from different combinations additive genetic variance of parasitoids for the three species model. The blue dashed line represents the parasitoid that is introduced first, and the red dotted line represents the parasitoid that is introduced second. "AgP" is the additive genetic variance of the first parastitoid, and "AgW" is the additive genetic variance of the second parasitoid.

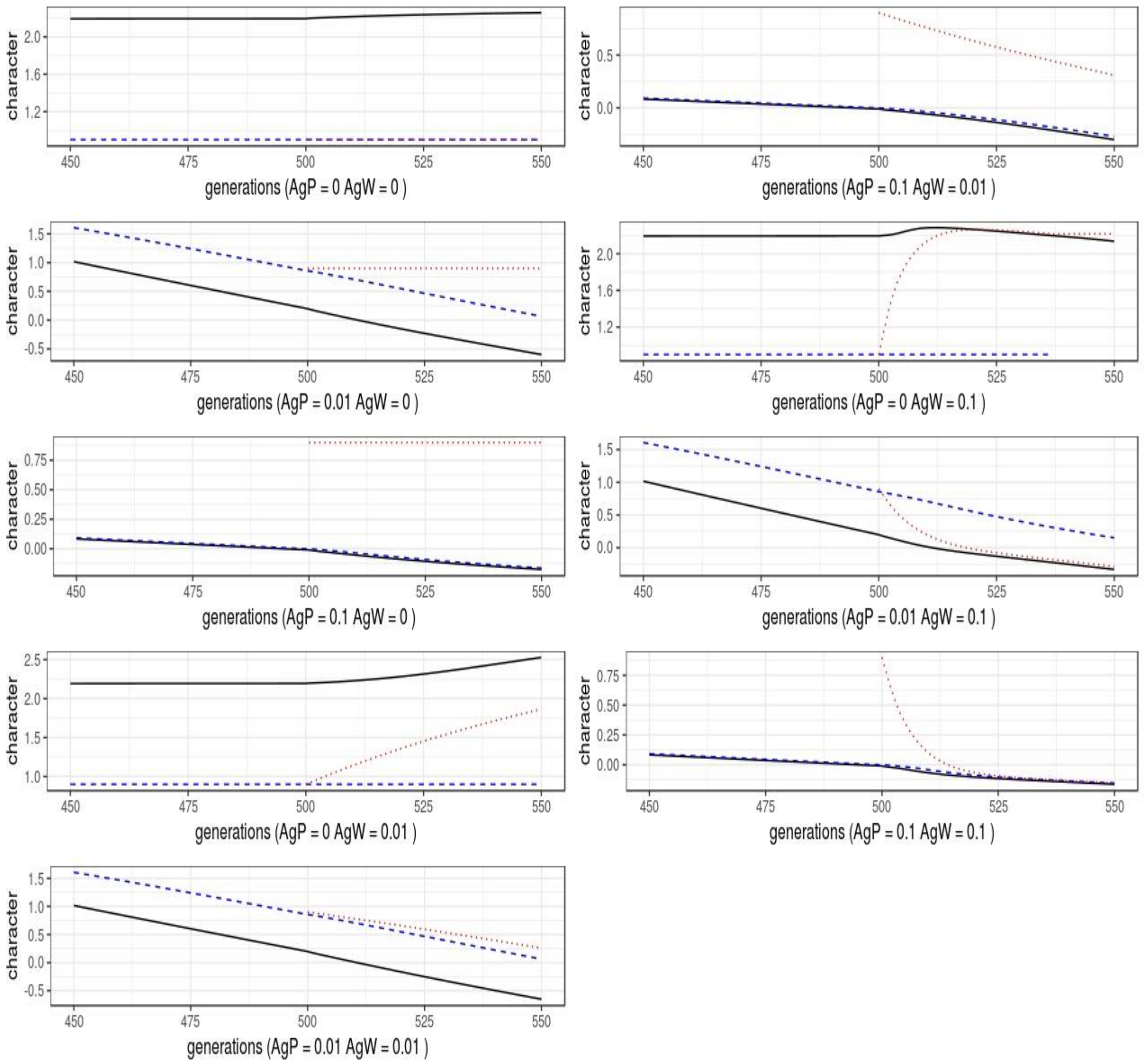

Fig L. Character value over time resulting from different combinations additive genetic variance of parasitoids for the three species model. The blue dashed line represents the parasitoid that is introduced first, and the red dotted line represents the parasitoid that is introduced second. "AgP" is the additive genetic variance of the first parastitoid, and "AgW" is the additive genetic variance of the second parasitoid.
